# Supplementary material for: Epigenetic and molecular coordination between HDAC2 and SMAD3-SKI regulates essential brain tumour stem cell characteristics
Source: Nat Commun. 2023 Aug 19;14:5051. doi: 10.1038/s41467-023-40776-y (PMC10439933; doi:10.1038/s41467-023-40776-y)
Supplement: Supplementary file 1 — Supplementary Information [file 41467_2023_40776_MOESM1_ESM.pdf]

Supplementary Information

a

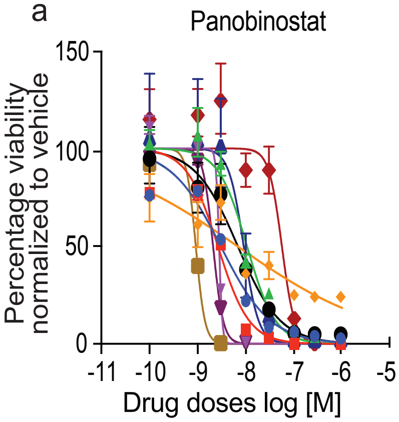

	IC50 (M)
BT50	3.000e-009
BT67	2.625e-009
BT147	1.053e-008
BT69	~2.787e-009
BT89	6.635e-009
BT94	6.792e-009
BT189	9.138e-010
BT248	9.011e-009
BT48	2.088e-009
HFA	5.584e-008

b

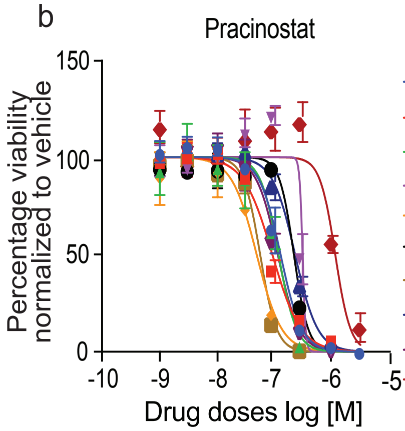

	IC50 (M)
BT50	1.236e-007
BT67	8.914e-008
BT147	1.140e-007
BT69	~2.982e-007
BT89	4.771e-008
BT94	2.104e-007
BT189	5.480e-008
BT248	2.207e-007
BT48	1.107e-007
HFA	1.095e-006

c

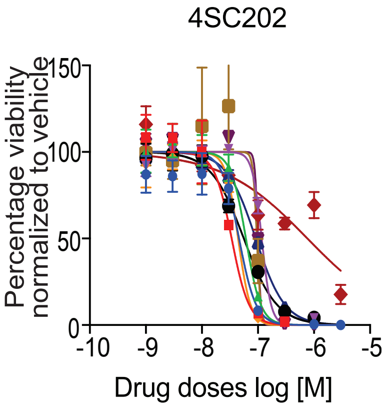

	IC50 (M)
BT50	4.599e-008
BT67	3.395e-008
BT147	6.092e-008
BT69	1.167e-007
BT89	4.430e-008
BT94	5.596e-008
BT189	~9.579e-008
BT248	9.128e-008
BT48	~9.987e-008
HFA	7.625e-007

d

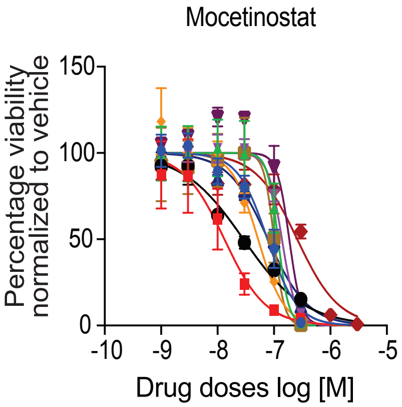

	IC50 (M)
BT50	7.668e-008
BT67	1.324e-008
BT147	1.181e-007
BT69	1.384e-007
BT89	5.235e-008
BT94	2.882e-008
BT189	1.006e-007
BT248	7.836e-008
BT48	1.868e-007
HFA	2.582e-007

e

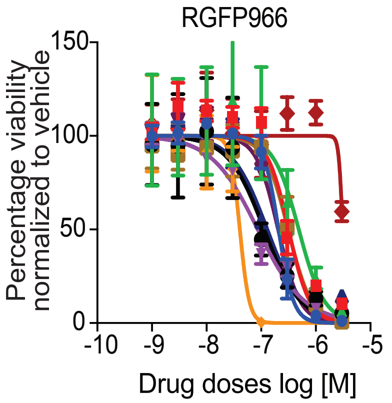

	IC50 (M)
BT50	2.080e-007
BT67	3.177e-007
BT147	4.874e-007
BT69	8.654e-008
BT89	4.064e-008
BT94	1.130e-007
BT189	3.106e-007
BT248	1.290e-007
BT48	2.043e-007
HFA	~3.096e-006

f

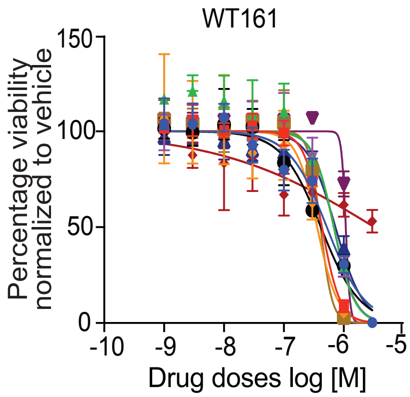

	IC50 (M)
BT50	5.229e-007
BT67	4.339e-007
BT147	6.838e-007
BT69	6.863e-007
BT89	3.607e-007
BT94	4.152e-007
BT189	4.103e-007
BT248	7.279e-007
BT48	~1.082e-006
HFA	3.259e-006

Supplementary Figure 1: a-f Screening of pan- and Class I HDAC inhibitors in BTSC lines. Pan HDAC inhibitors, panobinostat and pracinostat, Class I and II specific HDAC inhibitors such as 4SC202, mocetinostat and HDAC3 specific inhibitors RGFP966 and HDAC6 inhibitor WT161 were screened in 9 BTSC cell lines and normal human fetal astrocytes (HFAs). Data represent mean values ± SD, n=3. Source data are provided in the source data file.

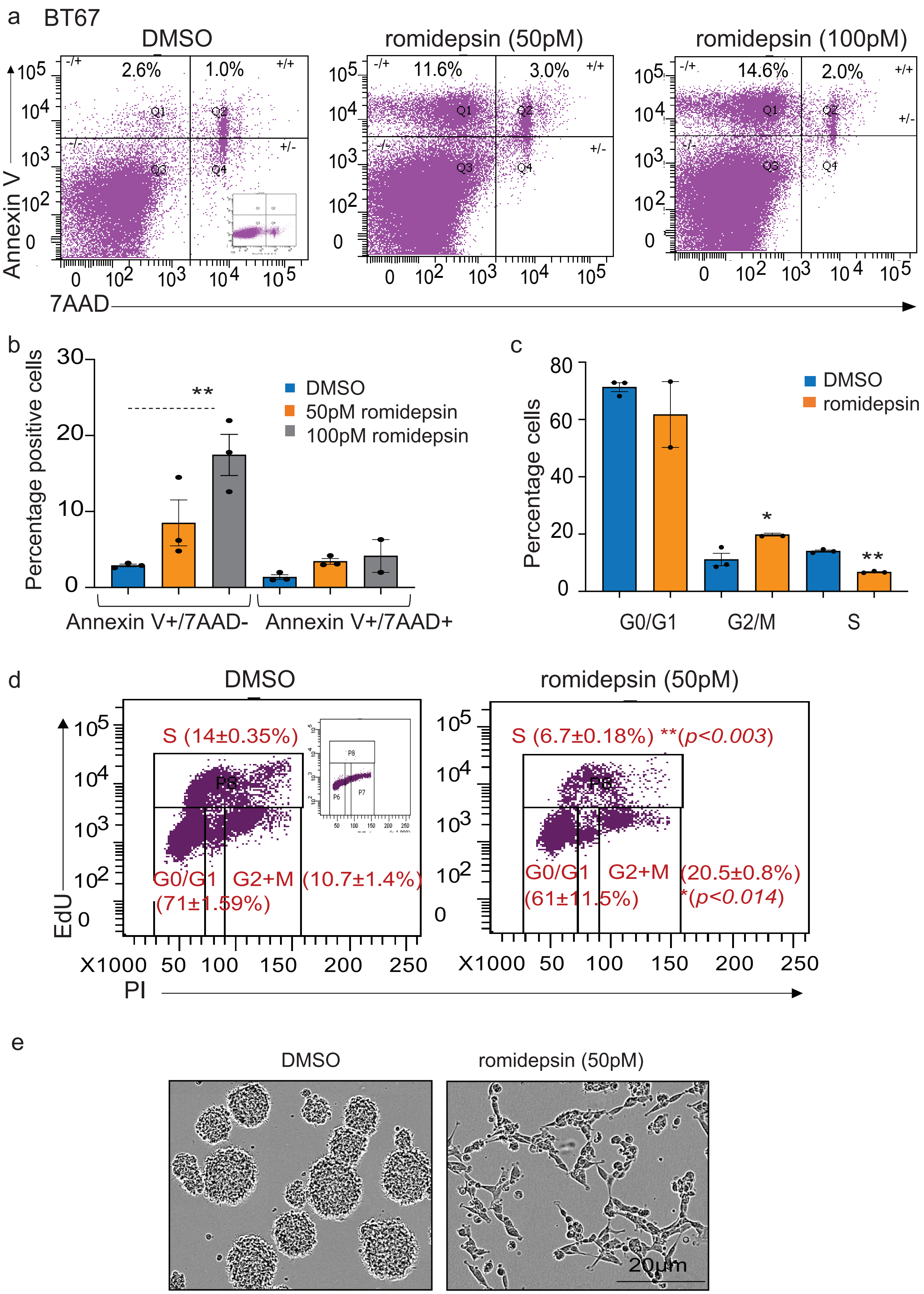
a BT67
DMSO
romidepsin (50pM)
romidepsin (100pM)
2.6%
1.0%
11.6%
3.0%
14.6%
2.0%
Q1 Q2 Q3 Q4
-/+ +/+ -/- +/-
Annexin V
7AAD
b
Percentage positive cells
**
DMSO
50pM romidepsin
100pM romidepsin
Annexin V+/7AAD-
Annexin V+/7AAD+
c
Percentage cells
DMSO
romidepsin
G0/G1
G2/M
S
*
**
d
DMSO
romidepsin (50pM)
S (14±0.35%)
S (6.7±0.18%) **(p<0.003)
G0/G1 (71±1.59%)
G2+M (10.7±1.4%)
G0/G1 (61±11.5%)
G2+M (20.5±0.8%) *(p<0.014)
P5 P6 P7 P8
EdU
X1000
PI
e
DMSO
romidepsin (50pM)
20µm

Supplementary Figure 2: a Representative flow plots for Annexin V staining following 5 days treatments with different doses of romidepsin in BT67 cells. **b** Quantification of Annexin V staining in BT67 cell line treated with vehicle control (DMSO), 50 and 100pM of doses of romidepsin, presented as percentage Annexin V and Annexin V/7AAD positive cells. Significance was determined using ANOVA (Dunnett's test), *p<0.05; data are represented as mean ± SEM; n=3. **c** EdU assays showing changes in different phases of cell cycle following treatment of BT67 cells with 100pM dose of romidepsin. Significance was determined using unpaired two-tailed t-test, *p<0.014, **p<0.003; data are represented as mean ± SEM; n=3. **d** Representative flow plots of EdU incorporation and DNA content (Propidium iodide (PI)) from DMSO vehicle control and romidepsin (50pM) treated BT67 cells. Inset shows 7AAD only for Annexin V and PI control only for EdU staining. Gating strategies are provided in Supp. Fig 22a, b. **e** Changes in cell morphology following treatment with romidepsin (50pM). n=3 Scale bar: 20μm. Source data are provided in the source data file.

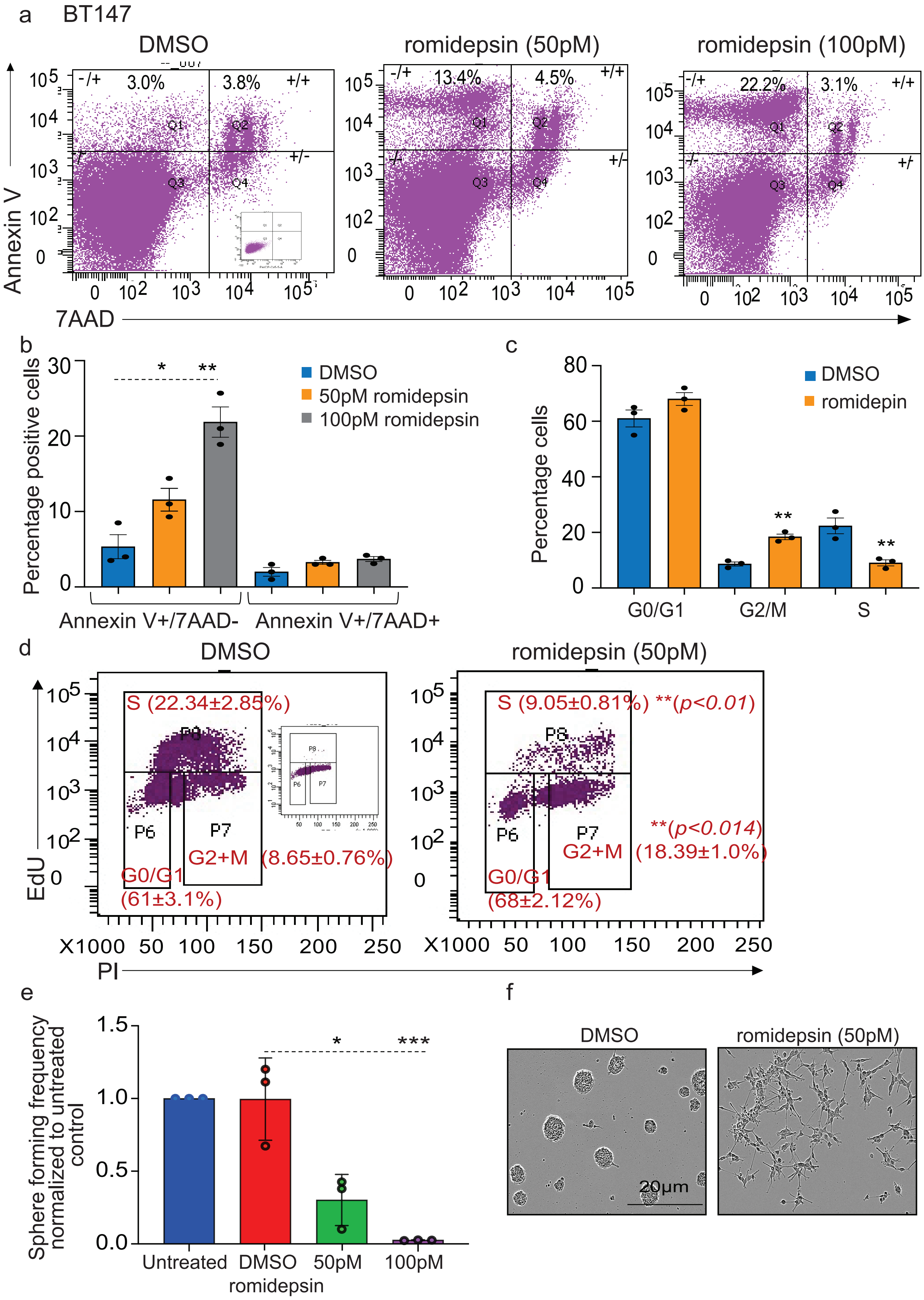

a BT147
DMSO
romidepsin (50pM)
romidepsin (100pM)
Annexin V
7AAD
-/+ 3.0% 3.8% +/+
-/+ 13.4% 4.5% +/+
-/+ 22.2% 3.1% +/+
Q1 Q2 Q3 Q4
b
Percentage positive cells
DMSO
50pM romidepsin
100pM romidepsin
Annexin V+/7AAD-
Annexin V+/7AAD+
c
Percentage cells
DMSO
romidepin
G0/G1
G2/M
S
d
DMSO
romidepsin (50pM)
EdU
PI
S (22.34±2.85%)
G2+M (8.65±0.76%)
G0/G1 (61±3.1%)
S (9.05±0.81%) **(p<0.01)
**(p<0.014)
G2+M (18.39±1.0%)
G0/G1 (68±2.12%)
P6 P7 P8
X1000 50 100 150 200 250
e
Sphere forming frequency normalized to untreated control
Untreated
DMSO
50pM
100pM
romidepsin
f
DMSO
romidepsin (50pM)
20µm

Supplementary Figure 3: a, b Quantification of Annexin V staining and representative flow plots of BT147 cell line treated with vehicle control (DMSO), 50 and 100pM doses of romidepsin for 5 days. Significance was determined using ANOVA (Dunnett's test), **p*<0.05, ***p*<0.01; data are represented as percentage Annexin V and Annexin V/7AAD positive cells mean ± SEM; n=3. **c, d** EdU cell cycle analysis and representative flow plots of EdU incorporation and DNA content (Propidium iodide (PI)) from DMSO vehicle control and romidepsin (50pM) treated BT147 cells. Significance was determined using unpaired two-tailed t-test, ***p*<0.014; data are represented as mean ± SEM; n=3. **e** Sphere forming frequency of BT147 cells following treatment with 50pM and 100pM doses of romidepsin as assessed with limiting dilution assays. Significance was determined using ANOVA (Dunnett's test), **p*<0.05, ****p*<0.001, Data represent mean ± upper and lower 95% confidence intervals, n=3. **f** Change in cell morphology following treatment with romidepsin (50pM). n=3 Scale bar: 20μm. Inset 7AAD only for Annexin V and PI control only for EdU staining. Gating strategies are provided in Supp. Fig 22a, b. Source data are provided in the source data file.

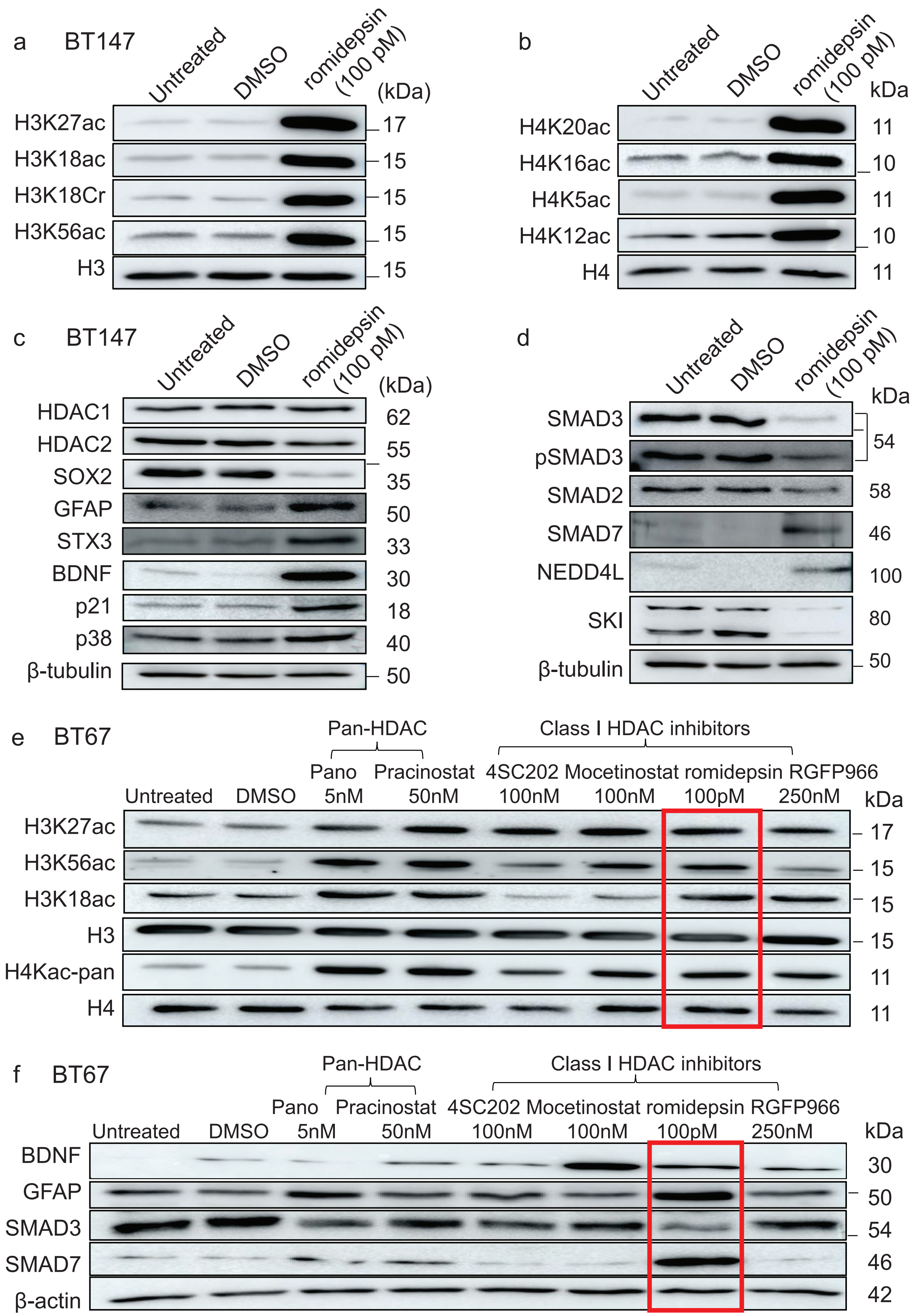
a BT147
Untreated
DMSO
romidepsin (100 pM)
(kDa)
H3K27ac 17
H3K18ac 15
H3K18Cr 15
H3K56ac 15
H3 15
b
Untreated
DMSO
romidepsin (100 pM)
kDa
H4K20ac 11
H4K16ac 10
H4K5ac 11
H4K12ac 10
H4 11
c BT147
Untreated
DMSO
romidepsin (100 pM)
(kDa)
HDAC1 62
HDAC2 55
SOX2 35
GFAP 50
STX3 33
BDNF 30
p21 18
p38 40
β-tubulin 50
d
Untreated
DMSO
romidepsin (100 pM)
kDa
SMAD3
pSMAD3
54
SMAD2 58
SMAD7 46
NEDD4L 100
SKI 80
β-tubulin 50
e BT67
Pan-HDAC
Class I HDAC inhibitors
Untreated
DMSO
Pano 5nM
Pracinostat 50nM
4SC202 100nM
Mocetinostat 100nM
romidepsin 100pM
RGFP966 250nM
kDa
H3K27ac 17
H3K56ac 15
H3K18ac 15
H3 15
H4Kac-pan 11
H4 11
f BT67
Pan-HDAC
Class I HDAC inhibitors
Untreated
DMSO
Pano 5nM
Pracinostat 50nM
4SC202 100nM
Mocetinostat 100nM
romidepsin 100pM
RGFP966 250nM
kDa
BDNF 30
GFAP 50
SMAD3 54
SMAD7 46
β-actin 42

Supplementary Figure 4: a, b Changes in global acetylation at H3 and H4 lysine residues in BT147 cells following 72 hours romidepsin (100pM) treatment relative to vehicle control, n=3. **c** Changes in protein levels of SOX2, GFAP, BDNF and the cell cycle regulators, CDKN1A (p21) and phospho-p38 in BT147 vehicle treated vs romidepsin treated cells, n=3. **d** Western blot analyses of components of the TGF-β pathway including total and phospho-SMAD3, the negative regulator proto-oncogene, SKI, the inhibitory SMAD protein, SMAD7, and the negative regulator of SMAD3, NEDD4L, in BT147 cells following romidepsin (100pM) treatment relative to vehicle control. n=3. **e** Screening of pan- and Class I and II specific inhibitors revealed that inhibition of class I HDAC, HDAC1 and 2 is sufficient to induce changes in global acetylation levels, n=3. **f** Evaluation of changes in protein levels of neuronal differentiation markers BDNF and GFAP; and TGF-β pathway related proteins, SMAD3 and SMAD7 following inhibition of different classes of HDACs using pan- and class- specific HDAC inhibitors in BT67 cells, n=3. - represents molecular weight markers (50, 37, 15 and 10). Source data are provided in the source data file.

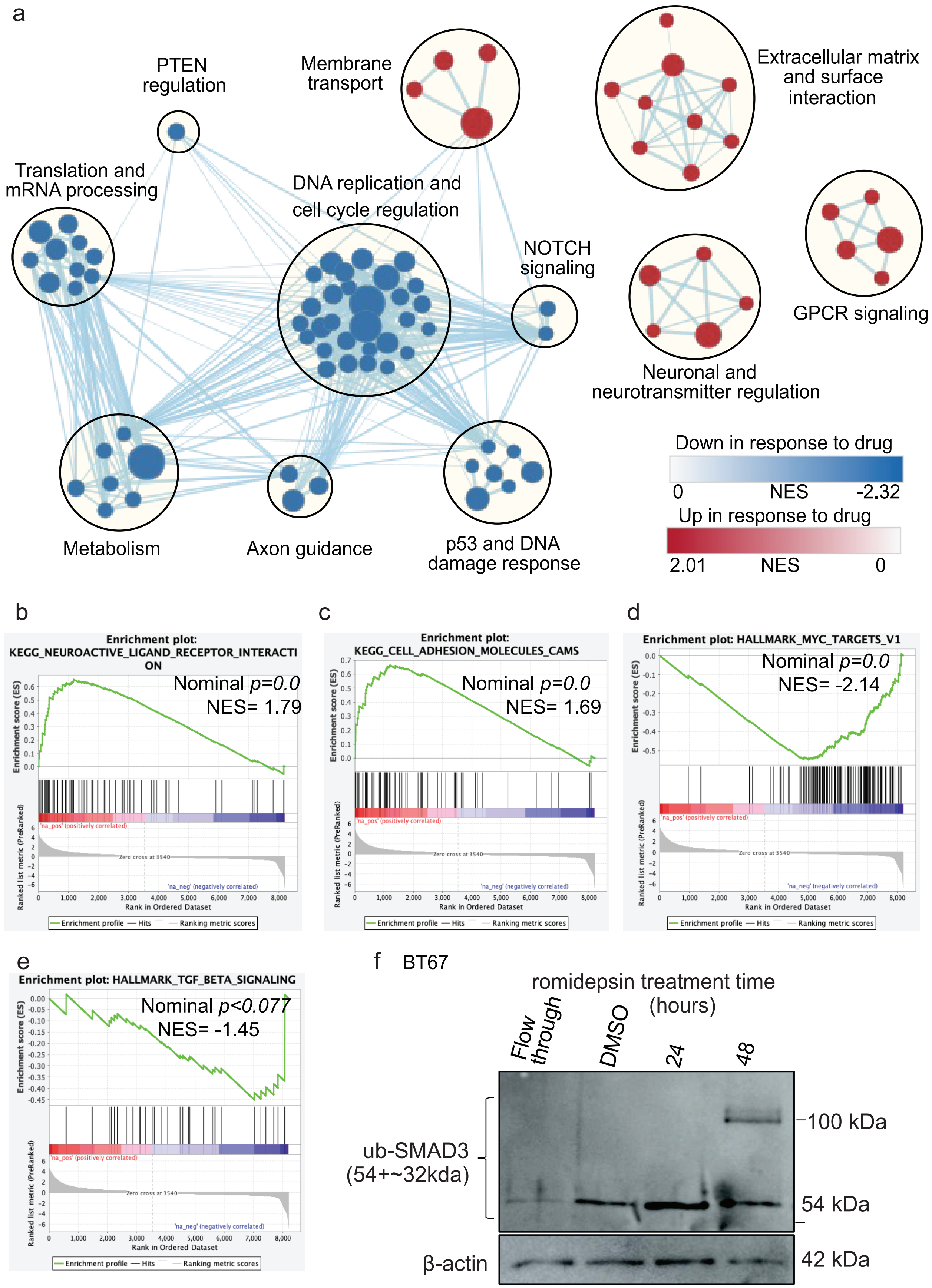
a
PTEN regulation
Membrane transport
Extracellular matrix and surface interaction
Translation and mRNA processing
DNA replication and cell cycle regulation
NOTCH signaling
GPCR signaling
Neuronal and neurotransmitter regulation
Metabolism
Axon guidance
p53 and DNA damage response
Down in response to drug
0 NES -2.32
Up in response to drug
2.01 NES 0
b
Enrichment plot: KEGG_NEUROACTIVE_LIGAND_RECEPTOR_INTERACTION
Nominal p=0.0
NES= 1.79
c
Enrichment plot: KEGG_CELL_ADHESION_MOLECULES_CAMS
Nominal p=0.0
NES= 1.69
d
Enrichment plot: HALLMARK_MYC_TARGETS_V1
Nominal p=0.0
NES= -2.14
e
Enrichment plot: HALLMARK_TGF_BETA_SIGNALING
Nominal p<0.077
NES= -1.45
Enrichment score (ES)
Ranked list metric (PreRanked)
Rank in Ordered Dataset
'na_pos' (positively correlated)
'na_neg' (negatively correlated)
Zero cross at 3540
Enrichment profile — Hits — Ranking metric scores
f BT67
romidepsin treatment time (hours)
Flow through
DMSO
24
48
ub-SMAD3 (54+~32kda)
100 kDa
54 kDa
β-actin
42 kDa

Supplementary Figure 5: a Gene set enrichment analysis of differentially expressed genes in romidepsin vs. vehicle control in BT67 cells. Significantly enriched gene sets (FDR<0.05) were visualized using Cytoscape with enriched maps and autoannotate plug-ins. n=3 **b-e** Enrichment plots for neuroactive ligand receptor interaction, cell adhesion molecules, MYC target genes and the TGF-β signaling with Nominal p value and nominal enrichment score (NES). **f** Polyubiquitination assay following different time course treatment with romidepsin vs. vehicle control, n=3. The ubiquitinated-SMAD3 protein representing an added molecular weight of approximately 32 kDa as per manufacturer's guidelines. – represents molecular weight markers (100 and 50). Source data are provided in the source data file.

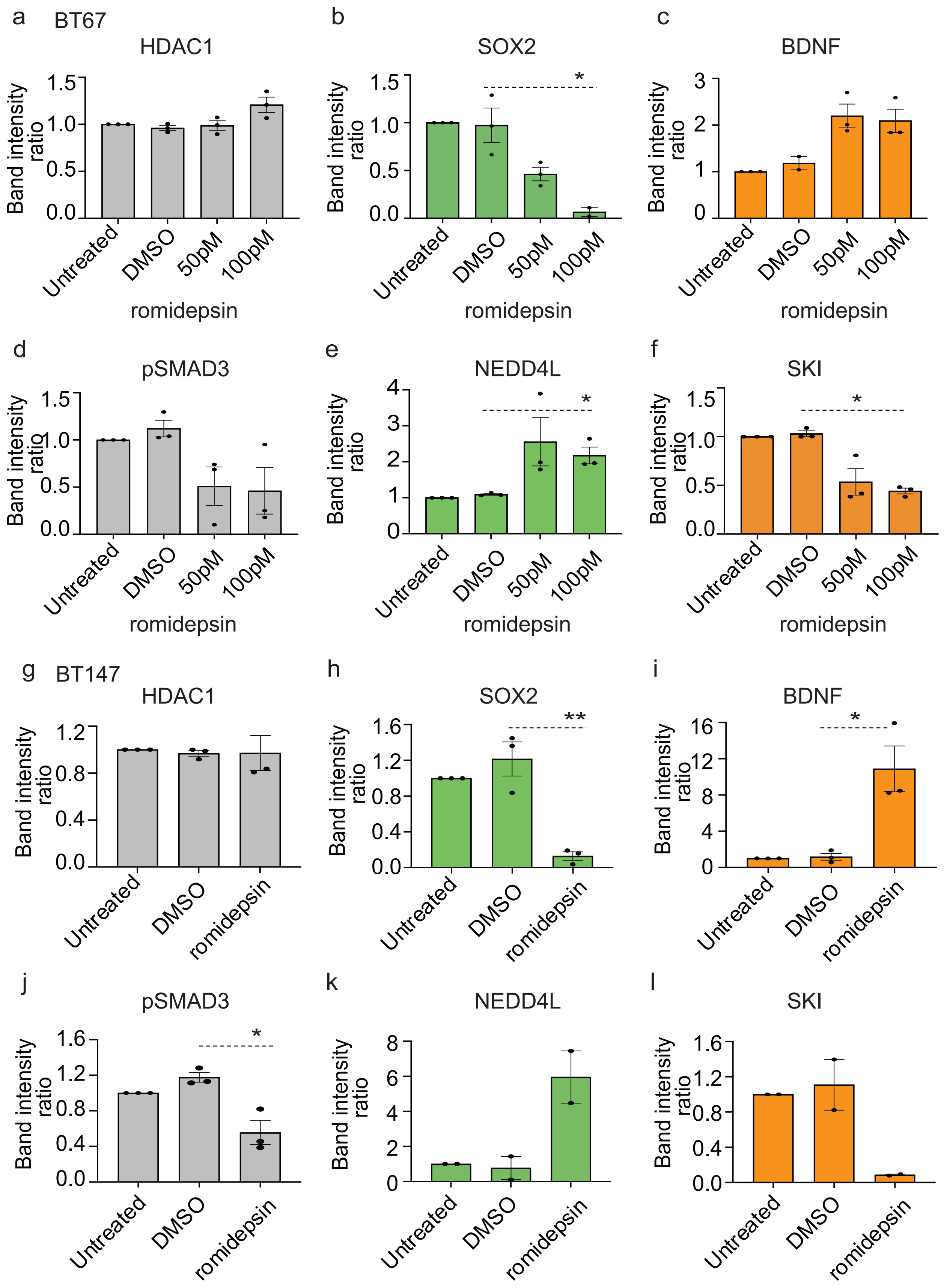

a
BT67
HDAC1
Band intensity ratio
1.5
1.0
0.5
0.0
Untreated
DMSO
50pM
100pM
romidepsin
b
SOX2
*
c
BDNF
3
2
1
0
d
pSMAD3
e
NEDD4L
4
3
2
1
0
f
SKI
g
BT147
1.2
0.8
0.4
0.0
h
1.6
**
i
16
12
8
4
j
k
l

Supplementary Figure 6: a-l Quantification of band intensity ratios for immunoblots presented in Fig 2a, b and Supp Fig 4c, d. Significance was determined using unpaired two-tailed t-test, *$p<0.05$, **$p<0.01$; data are represented as mean ± SEM, n=3. Source data are provided in the source data file.

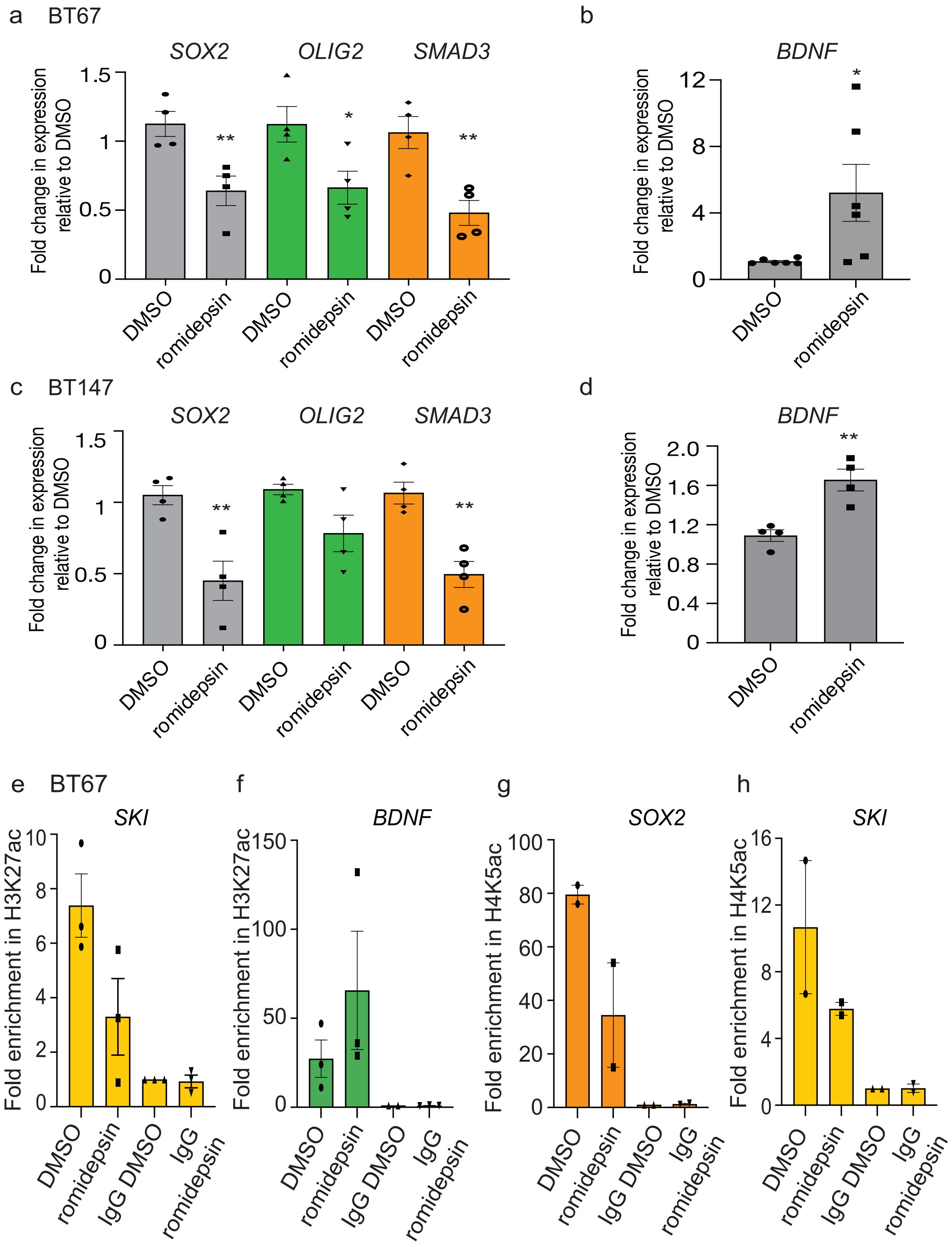
a BT67
SOX2
OLIG2
SMAD3
Fold change in expression relative to DMSO
1.5
1
0.5
0
**
*
**
DMSO
romidepsin
b
BDNF
*
12
8
4
0
c BT147
**
**
d
2.0
1.6
1.2
0.8
0.4
e BT67
SKI
Fold enrichment in H3K27ac
10
8
6
4
2
IgG DMSO
IgG romidepsin
f
150
100
50
g
Fold enrichment in H4K5ac
100
80
60
40
20
h
16
12

Supplementary Figure 7: a-d Quantitative real-time PCR validating the RNA-seq data showing fold change in the expression of *SOX2*, *OLIG2*, *SMAD3* and *BDNF* genes in romidepsin treated relative to the vehicle treated BT67 samples. Significance was determined using unpaired two-tailed t-test, **$p<0.01$, *$p<0.039$, **$p<0.0075$ (a), **$p<0.0078$, $p<0.06$, **$p<0.003$ (c), *$p<0.037$ (b), **$p<0.0041$; data are represented as fold change in expression mean values ± SEM; n=4. **e, f** ChIP-PCR validation of ChIP-seq data showing changes in the levels of the H3K27ac mark at the 5’ regulatory region of *SKI* and *BDNF* genes. Significance was determined using unpaired two-tailed t-test; data are represented as fold enrichment mean values ± SEM; n=3. **g, h** Assessment of changes in the H4K5ac mark, by ChIP-PCR, at the 5’ region of *SOX2* and *SKI* genes. Significance was determined using unpaired two-tailed t-test; data are represented as fold enrichment mean values± SEM; n=3. Source data are provided in the source data file.

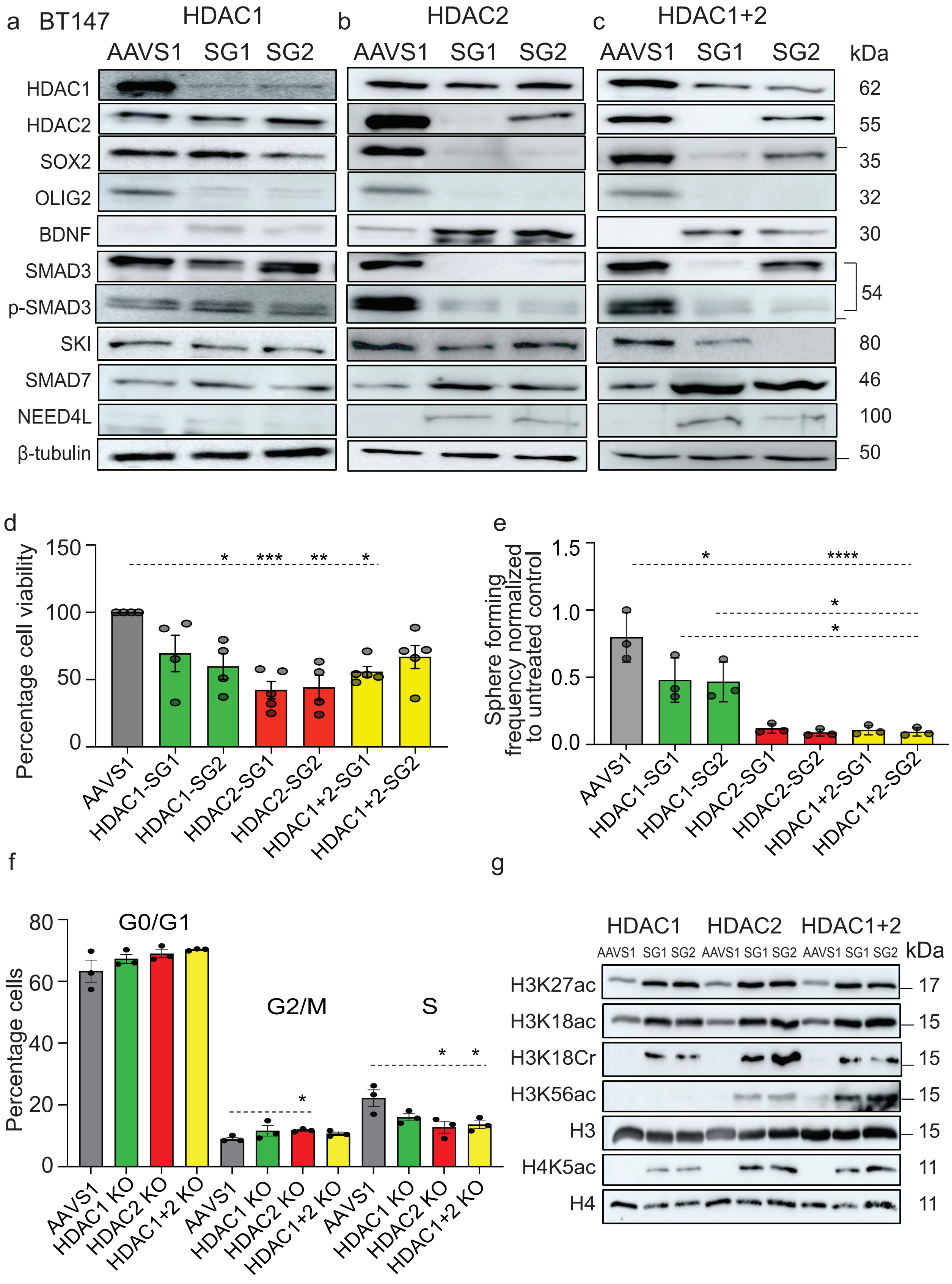

a BT147
HDAC1
AAVS1 SG1 SG2
b
HDAC2
AAVS1 SG1 SG2
c
HDAC1+2
AAVS1 SG1 SG2
kDa
HDAC1 62
HDAC2 55
SOX2 35
OLIG2 32
BDNF 30
SMAD3
p-SMAD3 54
SKI 80
SMAD7 46
NEED4L 100
β-tubulin 50
d
Percentage cell viability
150
100
50
0
* *** ** *
AAVS1
HDAC1-SG1
HDAC1-SG2
HDAC2-SG1
HDAC2-SG2
HDAC1+2-SG1
HDAC1+2-SG2
e
Sphere forming frequency normalized to untreated control
1.5
1.0
0.5
0.0
* ****
*
*
AAVS1
HDAC1-SG1
HDAC1-SG2
HDAC2-SG1
HDAC2-SG2
HDAC1+2-SG1
HDAC1+2-SG2
f
Percentage cells
80
60
40
20
0
G0/G1
G2/M
S
*
* *
AAVS1
HDAC1 KO
HDAC2 KO
HDAC1+2 KO
g
HDAC1 HDAC2 HDAC1+2
AAVS1 SG1 SG2 AAVS1 SG1 SG2 AAVS1 SG1 SG2
kDa
H3K27ac 17
H3K18ac 15
H3K18Cr 15
H3K56ac 15
H3 15
H4K5ac 11
H4 11

Supplementary Figure 8: a-c CRISPR-cas9 mediated knockout of *HDAC1* and *2* in BT147 cells using two independent guide RNAs for each gene and validation of target gene KOs. Western blots showing changes in protein levels of stem cell regulators; SOX2 and OLIG2, neuronal fate-specific marker; BDNF and the TGF-β pathway related proteins including total and phospho-SMAD3, SKI, SMAD7 and NEED4L in single and double *HDAC1/2* KO cells relative to the AAVS1 control cells (n=3, with two independent gRNAs). **d** Cell viability following single and double knockout of *HDAC1* and *2* in BT147 cells relative to AAVS1 cut control. Significance was determined using ANOVA (Tukey's test) at 95% confidence intervals, *p<0.05, **p<0.01, ***p<0.001; data are represented as mean ± SEM; n=3. **e** Sphere forming frequency of BT147 cells following single and double knockout of *HDAC1* and *2* in BT147 cells relative to AAVS1 cut control. Significance was determined using ANOVA (Tukey's test) at 95% confidence intervals, ***p<0.001, ****p<0.000; Data represent mean ± upper and lower 95% confidence intervals, n=3. **f** EdU analysis showing the effect of single and double knockout of HDAC1 and 2 on different cell cycle phases. Significance was determined using ANOVA (Dunnett's test) at 95% confidence intervals, *p<0.05; data are represented as mean ± SEM; n=3. Gating strategies are provided in Supp. Fig 22a. **g** Changes in Global acetylation levels of lysine residues of histones H3 and H4 in single and double *HDAC1* and *2* KO in BT147 cells (n=3). - represents molecular weight markers (50, 37, 15 and 10). Source data are provided in the source data file.

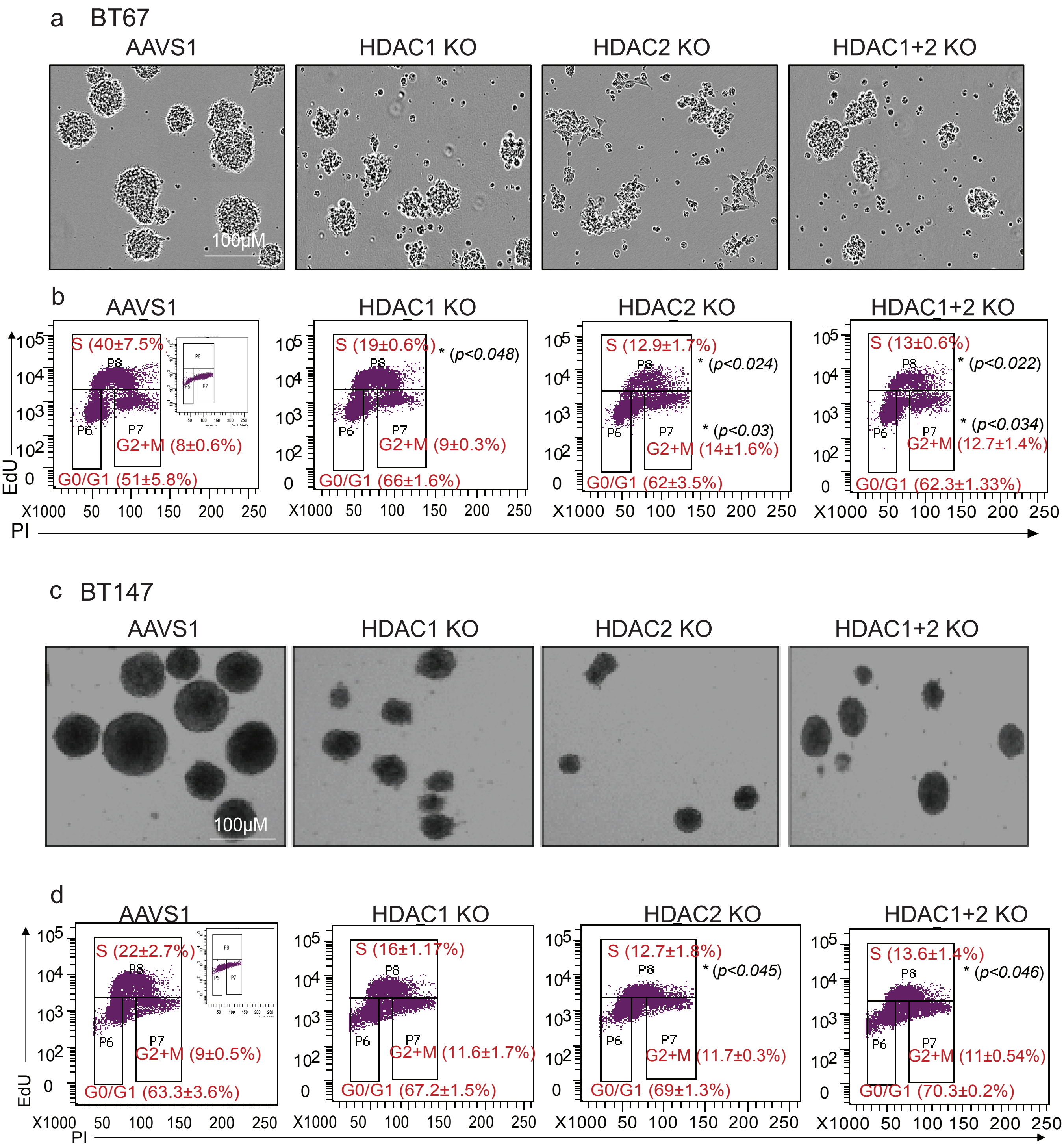

a BT67
AAVS1
HDAC1 KO
HDAC2 KO
HDAC1+2 KO
100μM
b
S (40±7.5%)
G2+M (8±0.6%)
G0/G1 (51±5.8%)
S (19±0.6%)
* (p<0.048)
G2+M (9±0.3%)
G0/G1 (66±1.6%)
S (12.9±1.7%)
* (p<0.024)
* (p<0.03)
G2+M (14±1.6%)
G0/G1 (62±3.5%)
S (13±0.6%)
* (p<0.022)
* (p<0.034)
G2+M (12.7±1.4%)
G0/G1 (62.3±1.33%)
EdU
PI
c BT147
AAVS1
HDAC1 KO
HDAC2 KO
HDAC1+2 KO
100μM
d
S (22±2.7%)
G2+M (9±0.5%)
G0/G1 (63.3±3.6%)
S (16±1.17%)
G2+M (11.6±1.7%)
G0/G1 (67.2±1.5%)
S (12.7±1.8%)
* (p<0.045)
G2+M (11.7±0.3%)
G0/G1 (69±1.3%)
S (13.6±1.4%)
* (p<0.046)
G2+M (11±0.54%)
G0/G1 (70.3±0.2%)
EdU
PI

Supplementary Figure 9: a, c Images of single and double *HDAC1* and *2* KO BT67 (**a**) and BT147 (**c**) cells. Scale bar: 100μm. **b, d** Representative flow plots showing changes in the phases of cell cycle in single and double *HDAC1* and *2* KO BT67 (**b**) and BT147 (**d)** cell lines (n=3). Inset PI control only. Gating strategies are provided in Supp. Fig 22a. Source data are provided in the source data file.

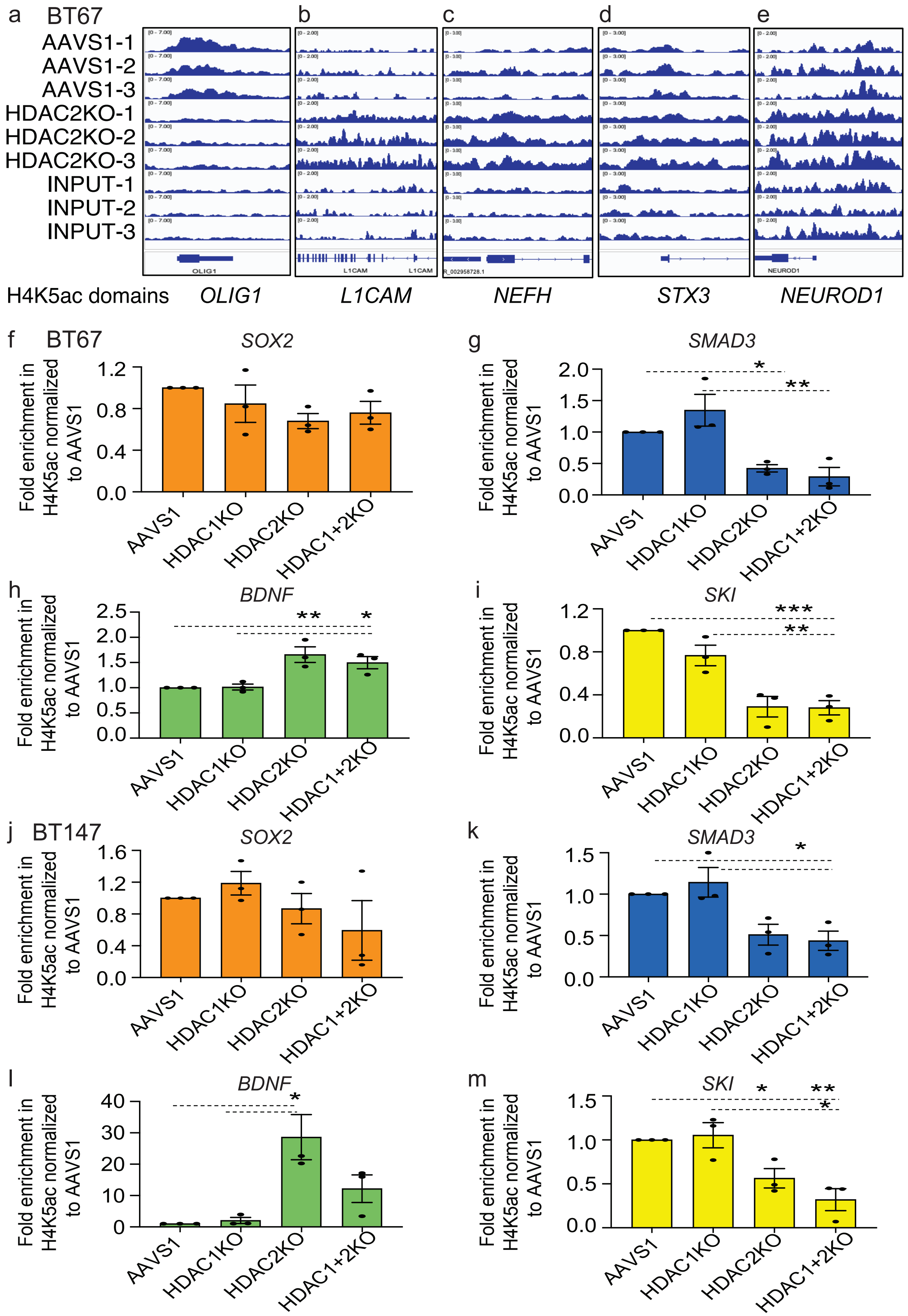

a BT67
AAVS1-1
AAVS1-2
AAVS1-3
HDAC2KO-1
HDAC2KO-2
HDAC2KO-3
INPUT-1
INPUT-2
INPUT-3
b
c
d
e
H4K5ac domains
OLIG1
L1CAM
NEFH
STX3
NEUROD1
f BT67
SOX2
g
SMAD3
h
BDNF
i
SKI
j BT147
SOX2
k
SMAD3
l
BDNF
m
SKI
Fold enrichment in H4K5ac normalized to AAVS1
AAVS1
HDAC1KO
HDAC2KO
HDAC1+2KO

Supplementary Figure 10: a-e ChIP-seq data showing changes in the H4K5ac domains at 5' regulatory regions of cell-fate related genes in *HDAC2* KO BTSCs relative to AAVS1 control cells ($p<0.05$, n=3). **f-m** Validation of ChIP-seq data by ChIP-qPCR for *SOX2*, *SMAD3*, *BDNF* and *SKI* genes in *HDAC1/2* single and double KO BTSCs. Significance was determined using ANOVA (Tukey's test) at 95% confidence intervals, *$p<0.05$, **$p<0.01$, ***$p<0.001$; data are represented as fold enrichment mean ± SEM; n=3. Source data are provided in the source data file.

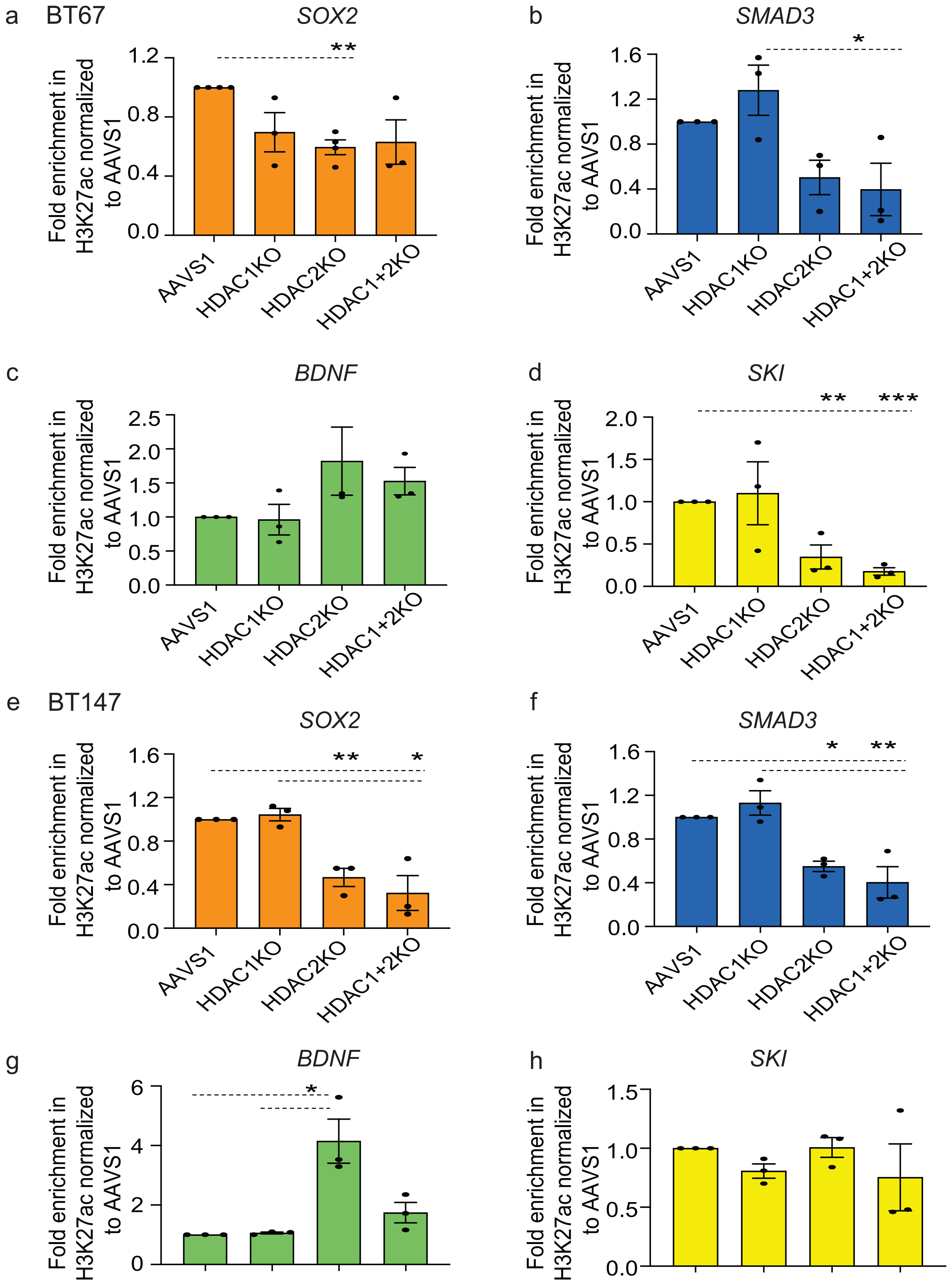

a
BT67
SOX2
**
Fold enrichment in H3K27ac normalized to AAVS1
1.2
0.8
0.4
0.0
AAVS1
HDAC1KO
HDAC2KO
HDAC1+2KO
b
SMAD3
*
Fold enrichment in H3K27ac normalized to AAVS1
1.6
1.2
0.8
0.4
0.0
AAVS1
HDAC1KO
HDAC2KO
HDAC1+2KO
c
BDNF
Fold enrichment in H3K27ac normalized to AAVS1
2.5
2.0
1.5
1.0
0.5
0.0
AAVS1
HDAC1KO
HDAC2KO
HDAC1+2KO
d
SKI
**
***
Fold enrichment in H3K27ac normalized to AAVS1
2.0
1.5
1.0
0.5
0.0
AAVS1
HDAC1KO
HDAC2KO
HDAC1+2KO
e
BT147
SOX2
**
*
Fold enrichment in H3K27ac normalized to AAVS1
1.6
1.2
0.8
0.4
0.0
AAVS1
HDAC1KO
HDAC2KO
HDAC1+2KO
f
SMAD3
*
**
Fold enrichment in H3K27ac normalized to AAVS1
1.6
1.2
0.8
0.4
0.0
AAVS1
HDAC1KO
HDAC2KO
HDAC1+2KO
g
BDNF
*
Fold enrichment in H3K27ac normalized to AAVS1
6
4
2
0
h
SKI
Fold enrichment in H3K27ac normalized to AAVS1
1.5
1.0
0.5
0.0

Supplementary Figure 11: a-h ChIP-qPCR showing changes in the fold enrichment of H3K27ac at 5' end of *SOX2*, *SMAD3*, *BDNF* and *SKI* genes in *HDAC1/2* single and double KO BTSCs. Significance was determined using ANOVA (Tukey's test) at 95% confidence intervals, *$p<0.05$, **$p<0.01$, ***$p<0.001$; data are represented as fold enrichment mean ± SEM; n=3. Source data are provided in the source data file.

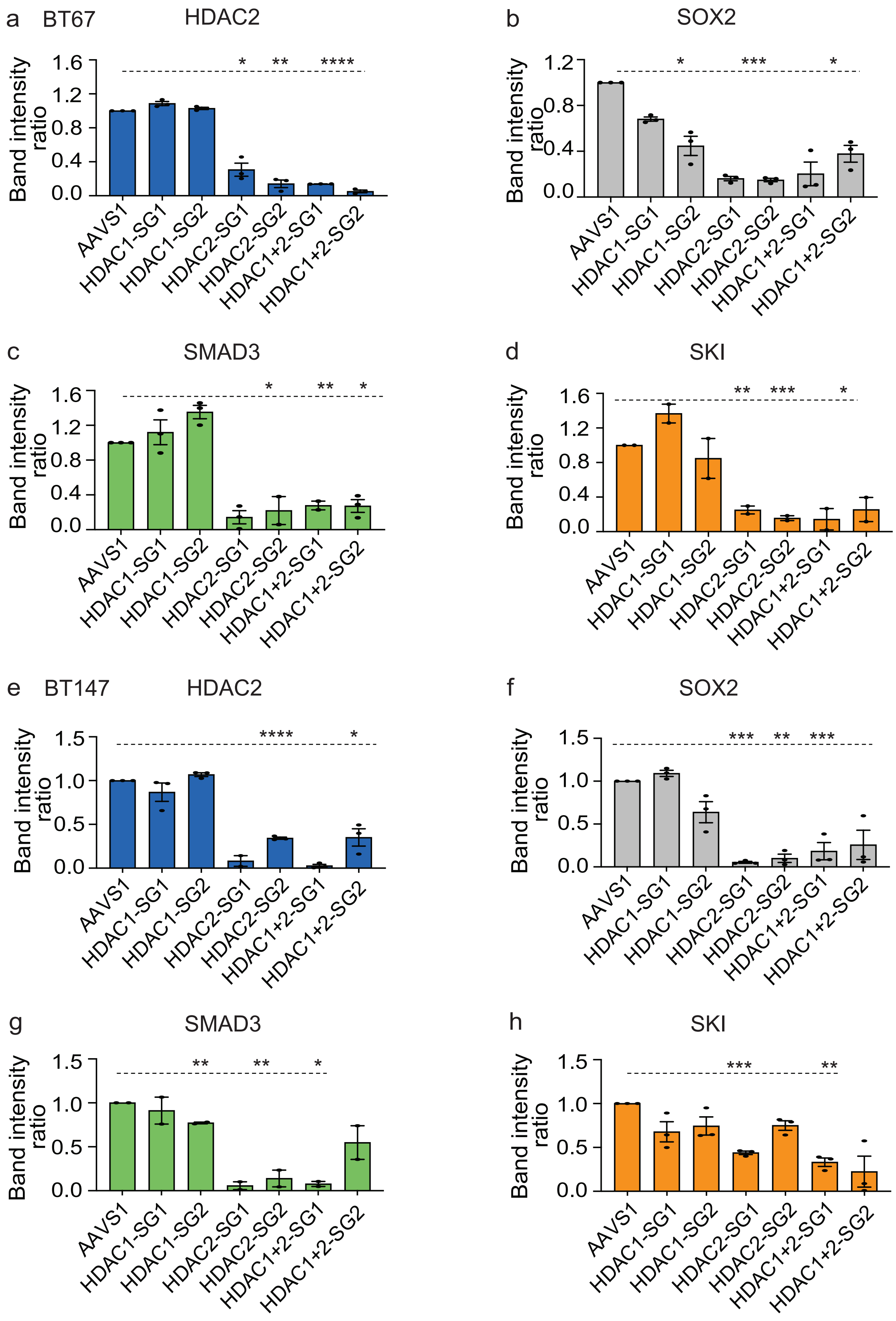

a
BT67
HDAC2
b
SOX2
c
SMAD3
d
SKI
e
BT147
HDAC2
f
SOX2
g
SMAD3
h
SKI
Band intensity ratio
AAVS1
HDAC1-SG1
HDAC1-SG2
HDAC2-SG1
HDAC2-SG2
HDAC1+2-SG1
HDAC1+2-SG2

Supplementary Figure 12: a-h Quantifications of band intensity ratios for immunoblots presented in Fig 3a-c and Supp Fig 8a-c. Significance was determined using ANOVA (Dunnett's test), **p*<0.05, ***p*<0.01, ****p*<0.001, *****p*<0.000; data are represented as mean ± SEM, n=3). Source data are provided in the source data file.

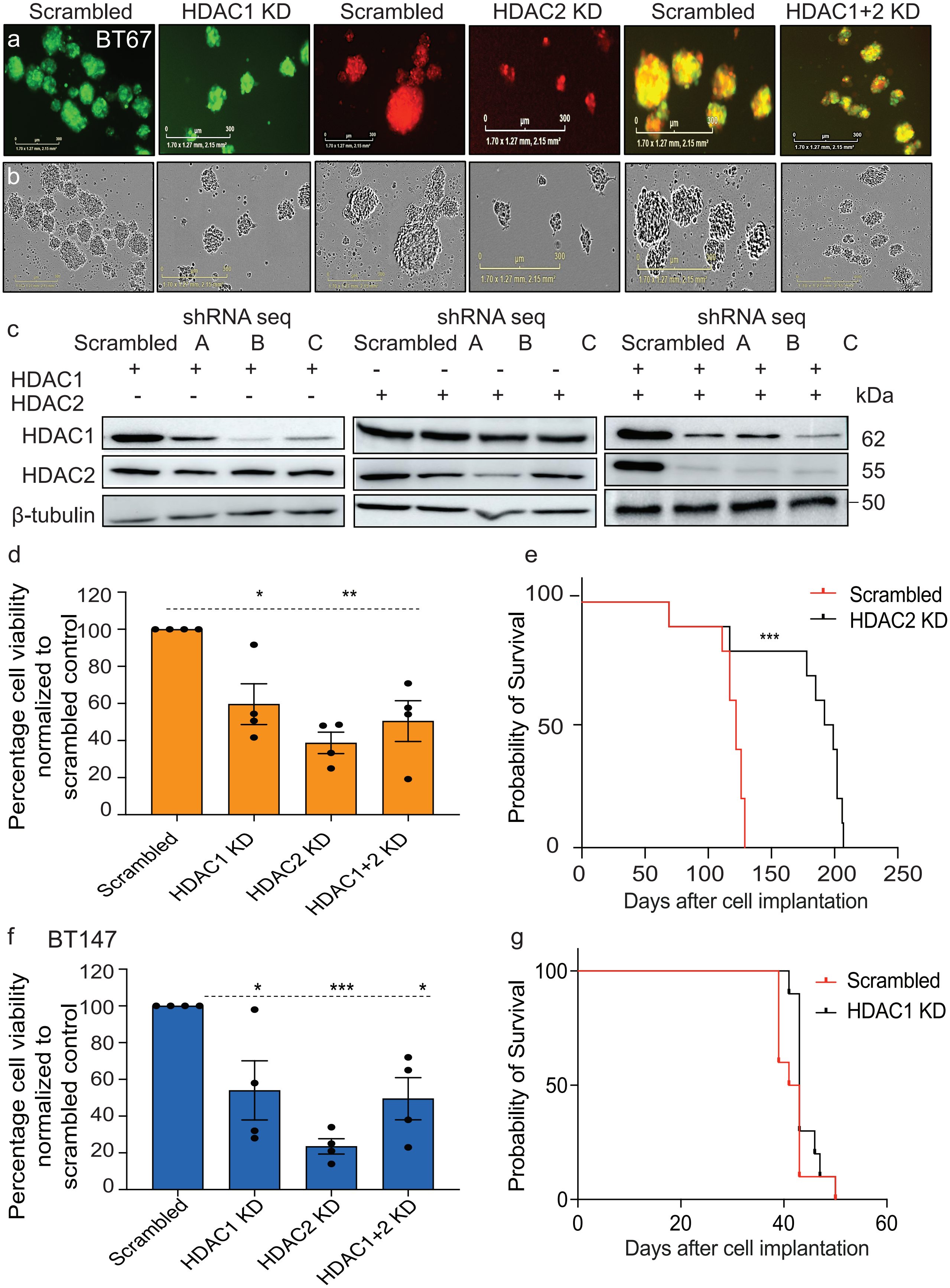

Scrambled
HDAC1 KD
Scrambled
HDAC2 KD
Scrambled
HDAC1+2 KD
a
BT67
b
c
shRNA seq
Scrambled A B C
HDAC1
HDAC2
kDa
HDAC1
HDAC2
β-tubulin
62
55
50
d
Percentage cell viability normalized to scrambled control
Scrambled
HDAC1 KD
HDAC2 KD
HDAC1+2 KD
e
Probability of Survival
Days after cell implantation
Scrambled
HDAC2 KD
***
f
BT147
g
HDAC1 KD

Supplementary Figure 13: a, b Fluorescent and brightfield images of shRNA mediated single and double KD of HDAC1 and HDAC2 in BT67 cells along with their respective scrambled control images. (Reporter gene; GFP for HDAC1 KD, mCherry for HDAC2 KD and double KD generated yellow fluorescence). Scale bar: 300μm. (n=3) **c** Validation of single and double KD of HDAC1 and 2 Western blot in BT67 cells. **d, f** Changes in cell viability following shRNA mediated single and double knockdown (KD) of HDAC1 and 2 in BT67 and BT147 cells relative to their respective scrambled controls. Significance was determined using unpaired two-tailed t-test, *p<0.05, **p<0.01, ***p<0.001; data are represented as mean ± SEM; n=3. **e, g** Kaplan-Meier survival curves for mice orthotopically xenograted with HDAC2 KD BT67(e) and HDAC1 KD BT147 (g) cells compared to mice xenografted with their respective scrambled control cells (Log-rank (Mantel-Cox) method, ***p<0.0011, n=10). - represents molecular weight markers (50). Source data are provided in the source data file.

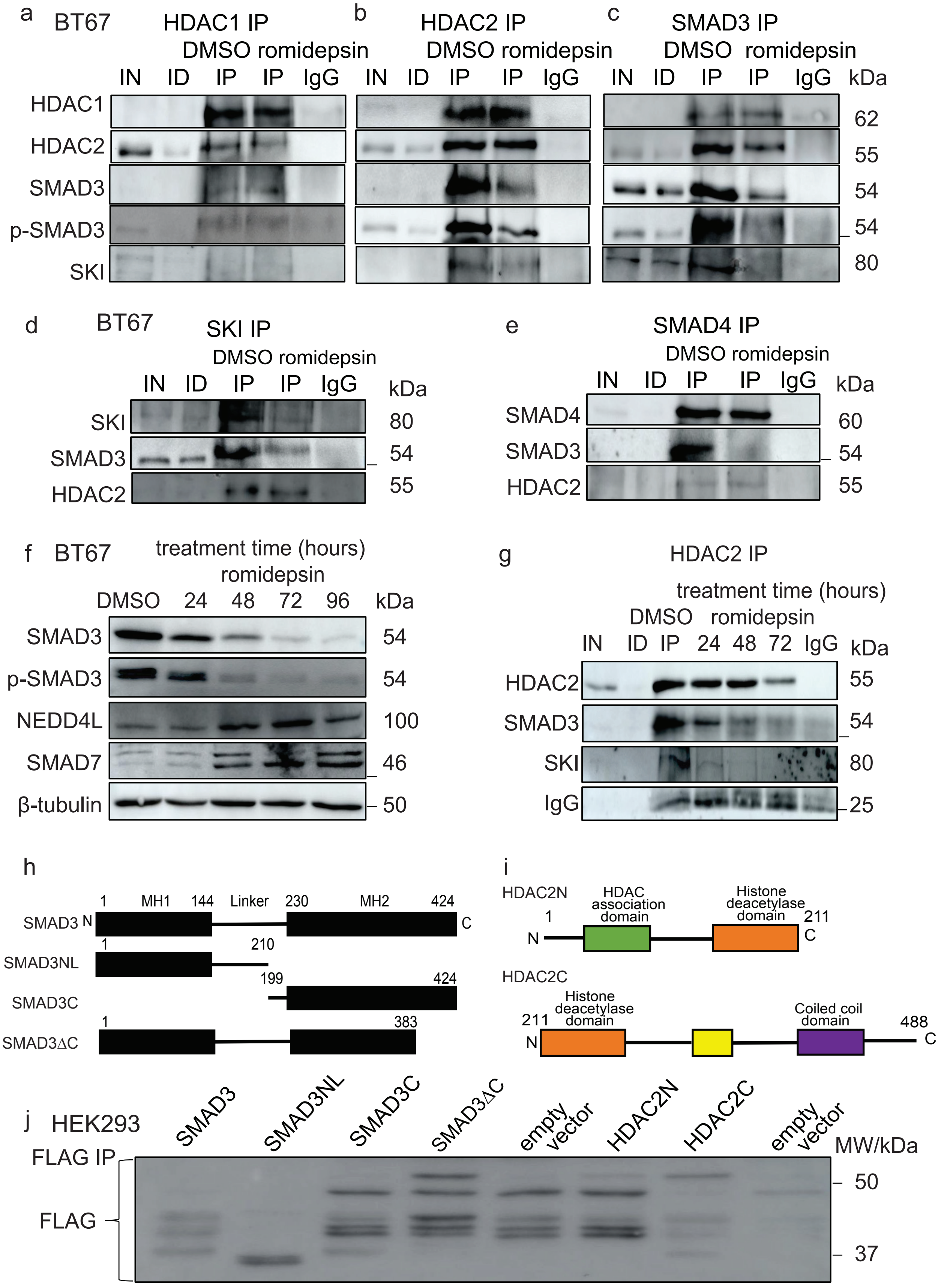

a BT67 HDAC1 IP
DMSO romidepsin
IN ID IP IP IgG
HDAC1
HDAC2
SMAD3
p-SMAD3
SKI
b HDAC2 IP
DMSO romidepsin
IN ID IP IP IgG
c SMAD3 IP
DMSO romidepsin
IN ID IP IP IgG kDa
62
55
54
54
80
d BT67 SKI IP
DMSO romidepsin
IN ID IP IP IgG kDa
SKI 80
SMAD3 54
HDAC2 55
e SMAD4 IP
DMSO romidepsin
IN ID IP IP IgG kDa
SMAD4 60
SMAD3 54
HDAC2 55
f BT67 treatment time (hours)
romidepsin
DMSO 24 48 72 96 kDa
SMAD3 54
p-SMAD3 54
NEDD4L 100
SMAD7 46
β-tubulin 50
g HDAC2 IP
treatment time (hours)
DMSO romidepsin
IN ID IP 24 48 72 IgG kDa
HDAC2 55
SMAD3 54
SKI 80
IgG 25
h
SMAD3 N 1 MH1 144 Linker 230 MH2 424 C
SMAD3NL 1 210
SMAD3C 199 424
SMAD3ΔC 1 383
i
HDAC2N
1 HDAC association domain Histone deacetylase domain 211
N C
HDAC2C
211 Histone deacetylase domain Coiled coil domain 488
N C
j HEK293
FLAG IP
FLAG
SMAD3
SMAD3NL
SMAD3C
SMAD3ΔC
empty vector
HDAC2N
HDAC2C
empty vector
MW/kDa
50
37

Supplementary Figure 14: a-e Co-immunoprecipitation assays using HDAC1, HDAC2, SMAD3, SKI and SMAD4 antibodies in dual crosslinked BT67 cells following 48 hours treatments with romidepsin and vehicle control (n=3). **f** Western blots showing changes in the protein levels of total and phospho-SMAD3, NEDD4L and SMAD7 in BT67 cells following time course treatments (24, 48, 72 and 96 hours) with romidepsin relative to the vehicle control (n=3). **g** Co-IP assays using HDAC1 antibody following time course treatment (24, 48 and 72 hours) with romidepsin relative to the vehicle control**. h-i** SMAD3 and HDAC2 mutant clone sequences. **j** Co-IP assay using anti-Flag antibody in HEK293T/17 cells expressing SMAD3 and HDAC2 mutant clones (n=3, biologically independent transductions). Input: 1% input was used for all the co-IP assays. IP-immunoprecipitation, ID-immunodepleted samples (1% ID sample was loaded on gel to check the antibody efficiency), IgG- antibody controls for non-specific pull down. - represents molecular weight markers (50, 37 and 25). Source data are provided in the source data file.

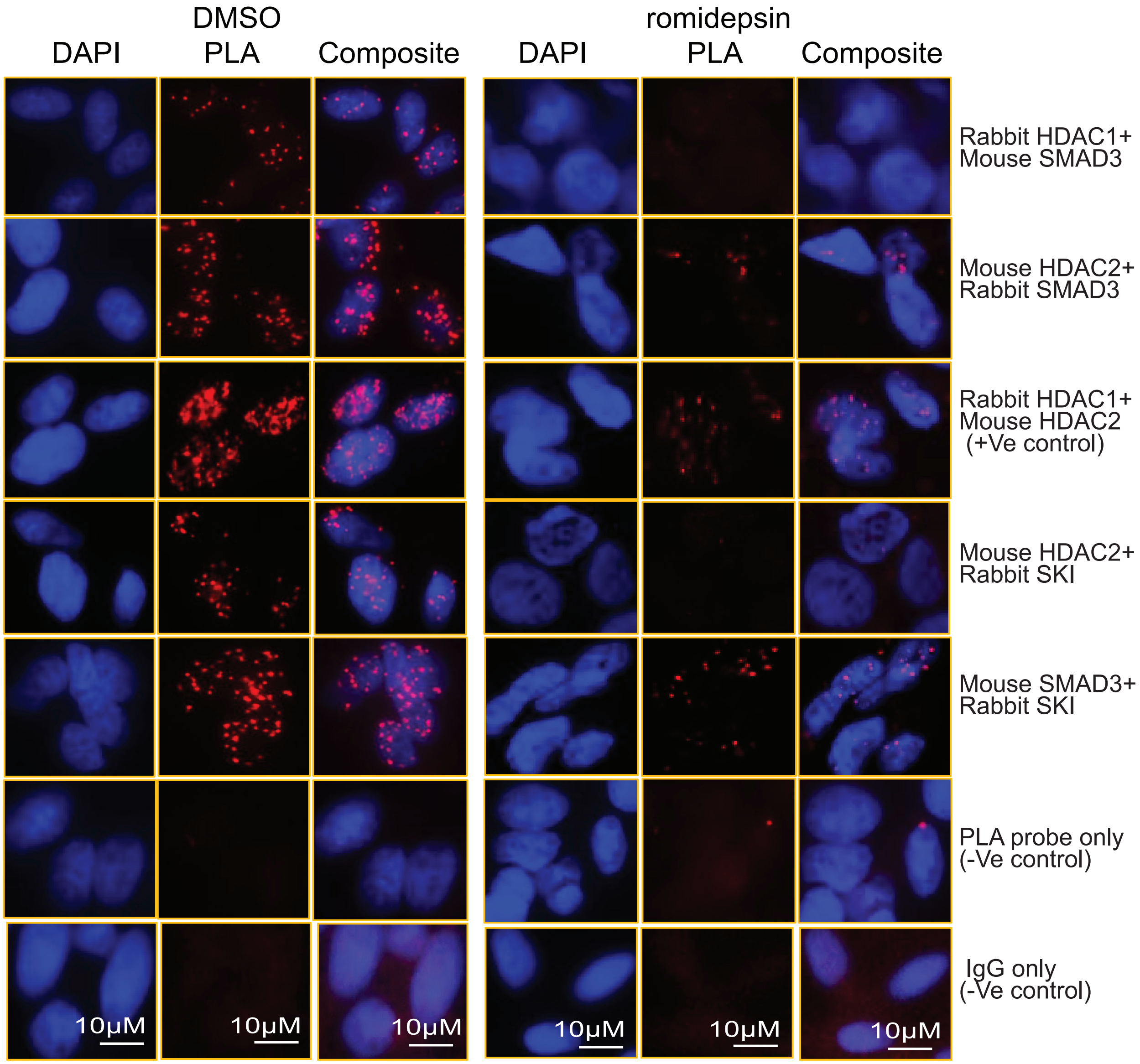
DMSO
DAPI
PLA
Composite
romidepsin
DAPI
PLA
Composite
Rabbit HDAC1+
Mouse SMAD3
Mouse HDAC2+
Rabbit SMAD3
Rabbit HDAC1+
Mouse HDAC2
(+Ve control)
Mouse HDAC2+
Rabbit SKI
Mouse SMAD3+
Rabbit SKI
PLA probe only
(-Ve control)
IgG only
(-Ve control)
10μM

Supplementary Figure 15: *In situ* proximity ligation assays (PLA) confirm protein-protein interactions between HDAC2 and the SMAD3-SKI proteins which were disrupted following 24 hours treatment with romidepsin relative to the vehicle control in BT67 cells visualized as a fluorescent signal. HDAC1/2 protein-protein interaction was used as positive control and PLA probe only and IgG only were used as negative controls for the assay. (n=2). DAPI- nuclear stain. Scale bar: 10μm.

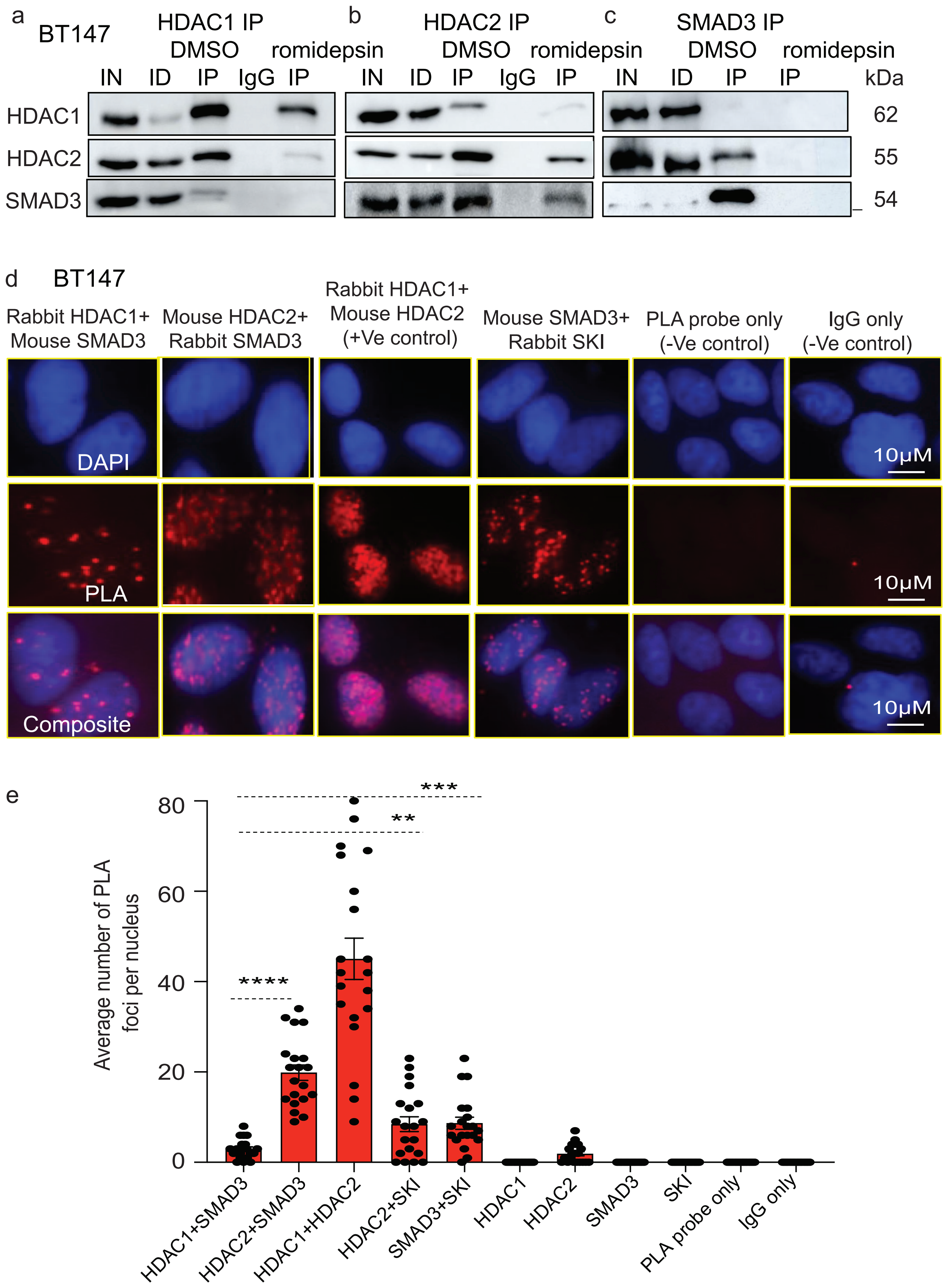
a
BT147
HDAC1 IP
DMSO
romidepsin
IN
ID
IP
IgG
IP
HDAC1
HDAC2
SMAD3
b
HDAC2 IP
DMSO
romidepsin
IN
ID
IP
IgG
IP
c
SMAD3 IP
DMSO
romidepsin
IN
ID
IP
IP
kDa
62
55
54
d
BT147
Rabbit HDAC1+ Mouse SMAD3
Mouse HDAC2+ Rabbit SMAD3
Rabbit HDAC1+ Mouse HDAC2 (+Ve control)
Mouse SMAD3+ Rabbit SKI
PLA probe only (-Ve control)
IgG only (-Ve control)
DAPI
PLA
Composite
10μM
10μM
10μM
e
Average number of PLA foci per nucleus
80
60
40
20
0
****
**
***
HDAC1+SMAD3
HDAC2+SMAD3
HDAC1+HDAC2
HDAC2+SKI
SMAD3+SKI
HDAC1
HDAC2
SMAD3
SKI
PLA probe only
IgG only

Supplementary Figure 16: a-c Co-IP assays using HDAC1, HDAC2 and SMAD3 antibodies in dual crosslinked BT147 cells following 72 hours treatment with romidepsin vs. vehicle control. Input: 1% input was used for all the co-IP assays. IP-immunoprecipitation, ID-immunodepleted samples (1% ID sample was loaded on gel to check the antibody efficiency), IgG- antibody controls for non-specific pull down (n=3). **d** Proximity ligation assays reproducing the similar findings in BT147 cells as seen in BT67 cells. (n=3) DAPI-nuclear stain. Scale bar: 20μm. **e** Quantitative representation of the average number of PLA foci counted in 30 nuclei/reaction in BT147 cells. Significance was determined using ANOVA (Tukey's test) at 95% confidence intervals, (**p<0.01, ***p<0.001, ****p<0.000, Data are represented as mean ± SEM; n=3). - represents molecular weight markers (50). Source data are provided in the source data file.

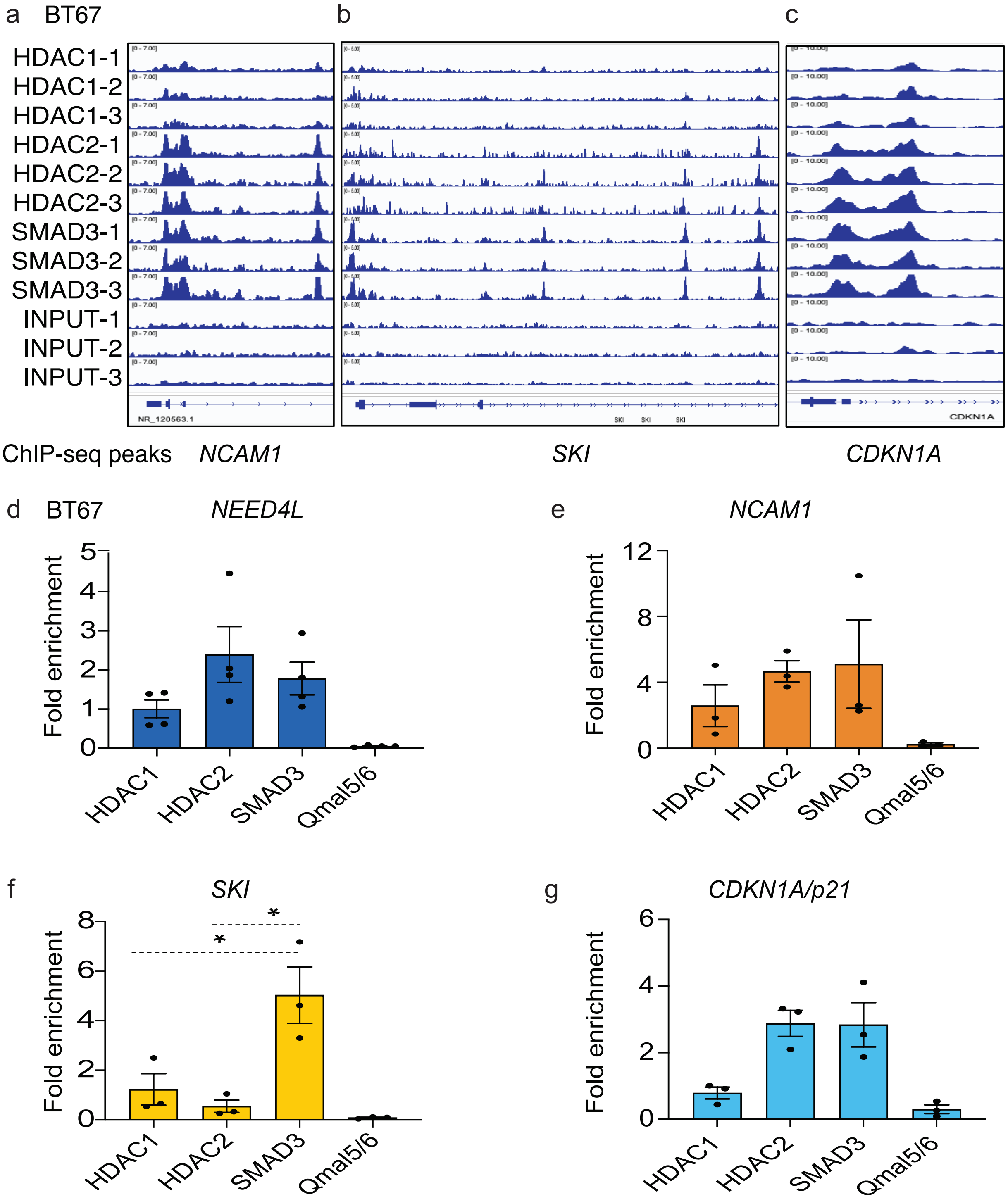

a BT67
b
c
HDAC1-1
HDAC1-2
HDAC1-3
HDAC2-1
HDAC2-2
HDAC2-3
SMAD3-1
SMAD3-2
SMAD3-3
INPUT-1
INPUT-2
INPUT-3
NR_120563.1
SKI
CDKN1A
ChIP-seq peaks
NCAM1
SKI
CDKN1A
d BT67
NEED4L
e
NCAM1
f
SKI
g
CDKN1A/p21
Fold enrichment
HDAC1
HDAC2
SMAD3
Qmal5/6
*

Supplementary Figure 17: a-c Gene tracks showing the enrichment levels of HDAC1/2 and SMAD3 within the gene regions of *NCAM1*, *SKI* and *CDKN1A* genes. **d-g** ChIP-qPCR validation of HDAC1/2 and SMAD3 ChIP-seq data for *NEED4L*, *NCAM1*, *SKI* and *CDKN1A* genes. Significance was determined using unpaired two-tailed t-test, *$p<0.05$; data are represented as fold enrichment mean ± SEM; n=3. Fold enrichment of HDAC1/2 and SMAD3 within the heterochromatin region QML5/6 was used as negative control for ChIP-qPCR. Source data are provided in the source data file.

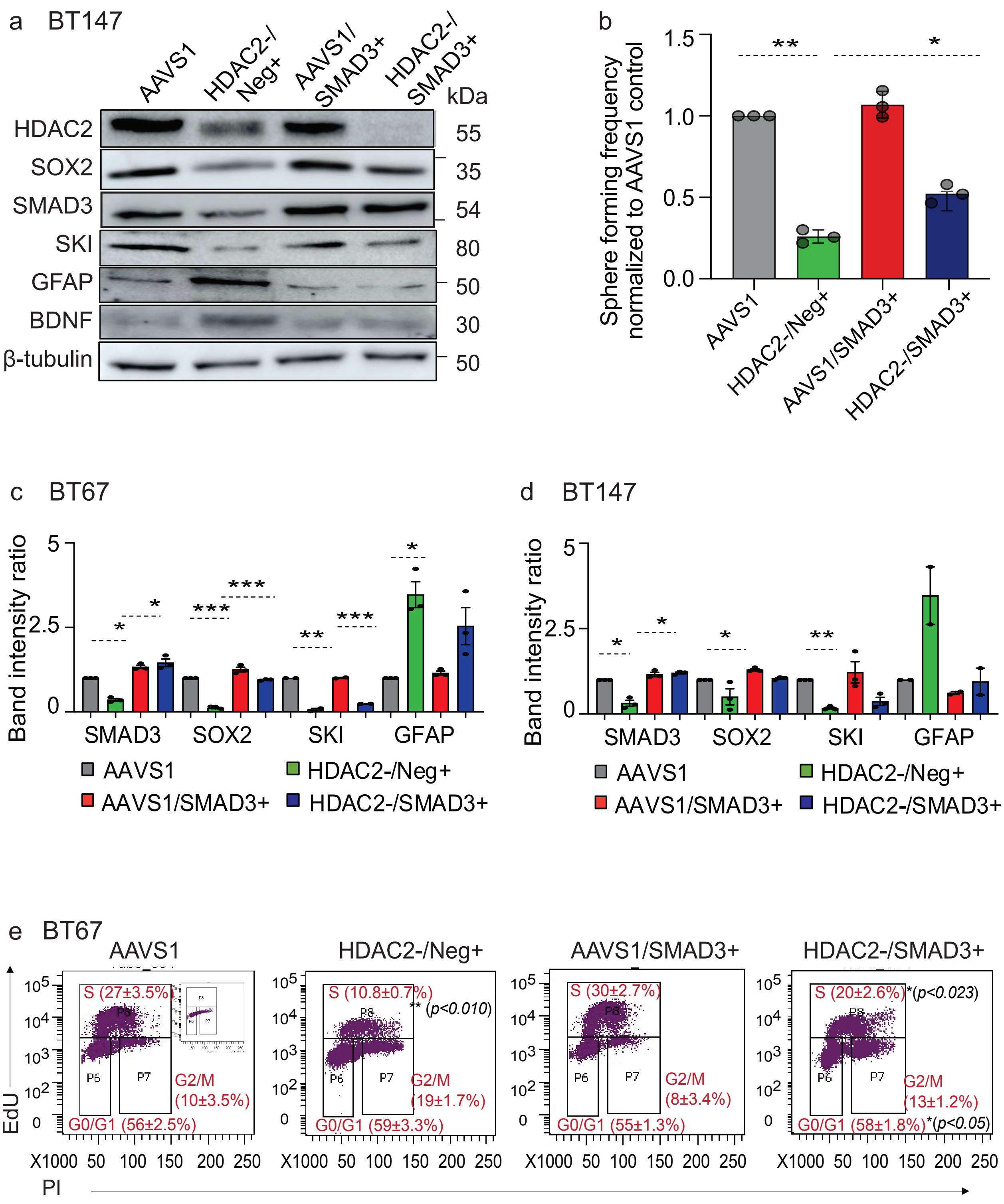
a BT147
AAVS1
HDAC2-/Neg+
AAVS1/SMAD3+
HDAC2-/SMAD3+
kDa
HDAC2 55
SOX2 35
SMAD3 54
SKI 80
GFAP 50
BDNF 30
β-tubulin 50
b
Sphere forming frequency normalized to AAVS1 control
1.5
1.0
0.5
0.0
**
*
AAVS1
HDAC2-/Neg+
AAVS1/SMAD3+
HDAC2-/SMAD3+
c BT67
Band intensity ratio
5
2.5
0
SMAD3
SOX2
SKI
GFAP
AAVS1
HDAC2-/Neg+
AAVS1/SMAD3+
HDAC2-/SMAD3+
d BT147
Band intensity ratio
5
2.5
0
SMAD3
SOX2
SKI
GFAP
AAVS1
HDAC2-/Neg+
AAVS1/SMAD3+
HDAC2-/SMAD3+
e BT67
AAVS1
HDAC2-/Neg+
AAVS1/SMAD3+
HDAC2-/SMAD3+
S (27±3.5%)
G2/M (10±3.5%)
G0/G1 (56±2.5%)
S (10.8±0.7%) ** (p<0.010)
G2/M (19±1.7%)
G0/G1 (59±3.3%)
S (30±2.7%)
G2/M (8±3.4%)
G0/G1 (55±1.3%)
S (20±2.6%) *(p<0.023)
G2/M (13±1.2%)
G0/G1 (58±1.8%) *(p<0.05)
P6
P7
P8
EdU
PI
X1000 50 100 150 200 250

Supplementary Figure 18: a Constitutive overexpression of SMAD3 in HDAC2 knockout and AAVS1 control BT147 cells (n=3 with two independent gRNAs). **b** Sphere forming frequency, measured following SMAD3 overexpression, in *HDAC2* KO and AAVS1 control BT147 cells. Significance was determined using ANOVA (Tukey's test) at 95% confidence intervals, **p*<0.05, ***p*<0.01; Data represent mean ± upper and lower 95% confidence intervals (n=3). **c, d** Quantification of band intensity ratios for immunoblots presented in Fig 6a and Supp Fig 18a. Significance was determined using ANOVA (Tukey's test) at 95% confidence intervals, **p*<0.05, ***p*<0.01, ****p*<0.001; data are represented as mean ± SEM, n=3. **e** Representative flow plots of EdU assays showing a mild rescue of cell proliferation in $HDAC2^{-}/SMAD3^{+}$ BT67 cells compared to $HDAC2^{-}/neg^{+}$ BT67 cells. Inset PI control only. Gating strategies are provided in Supp. Fig 22a. - represents molecular weight markers (50 and 37). Source data are provided in the source data file.

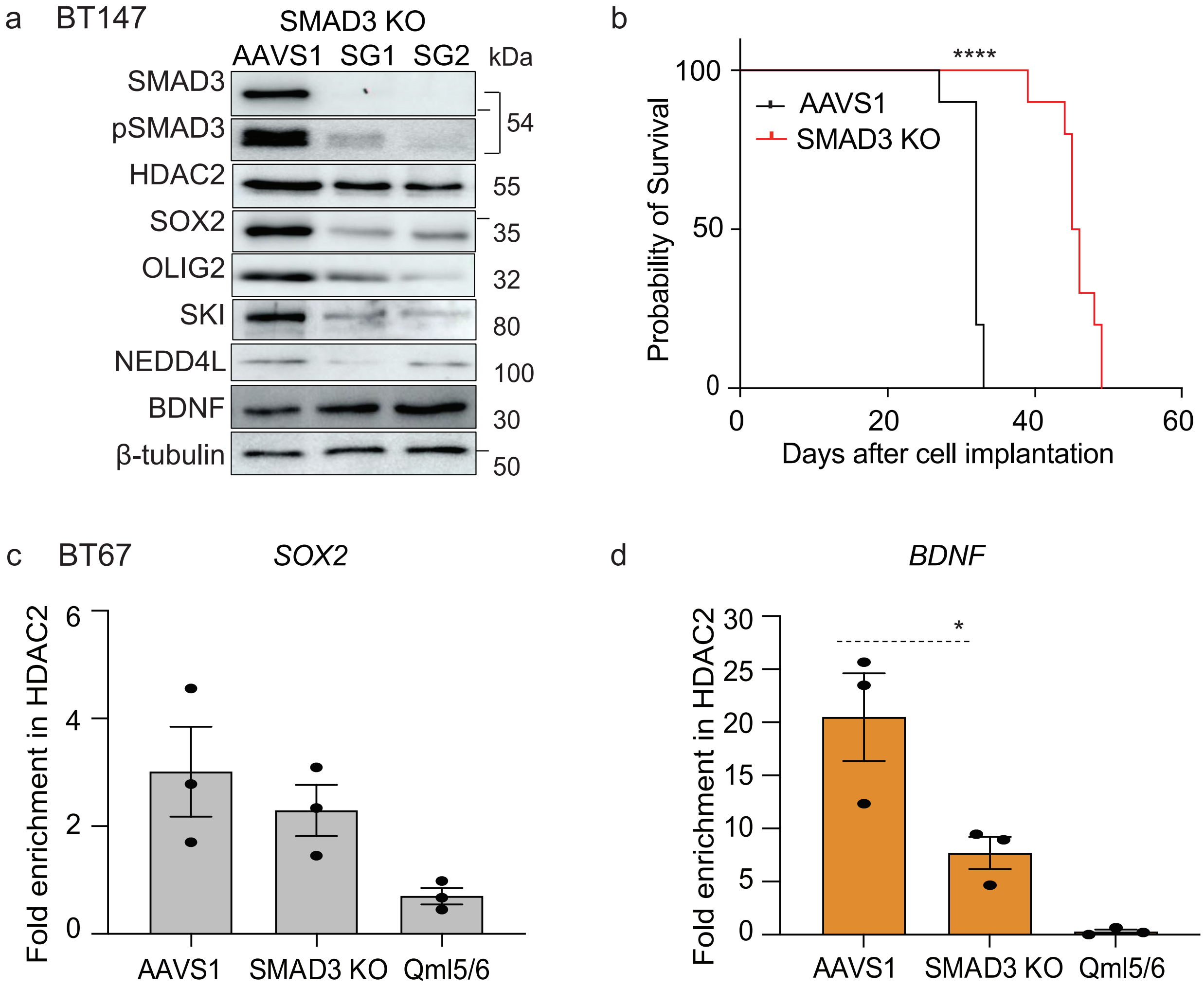
a
BT147
SMAD3 KO
AAVS1
SG1
SG2
kDa
SMAD3
pSMAD3
54
HDAC2
55
SOX2
35
OLIG2
32
SKI
80
NEDD4L
100
BDNF
30
β-tubulin
50
b
****
AAVS1
SMAD3 KO
Probability of Survival
Days after cell implantation
c
BT67
SOX2
Fold enrichment in HDAC2
AAVS1
SMAD3 KO
Qml5/6
d
BDNF
*
Fold enrichment in HDAC2
AAVS1
SMAD3 KO
Qml5/6

Supplementary Figure 19: a Western blot validation of CRISPR-cas9 mediated KO of *SMAD3* and its effect on HDAC2, SOX2, OLIG2, SKI, NEDD4L and BDNF protein levels in BT147 relative to AAVS1 control cells (n=3). **b** Kaplan-Meier survival curves for mice orthotopically xenografted with *SMAD3* KO BT147 cells compared to AAVS1 control mice (Log-rank (Mantel-Cox) method, ****p<0.0001, n=10). **c, d** ChIP-PCR showing changes in the enrichment levels of HDAC2 at the 5' regulatory region of *SOX2* and *BDNF* genes in *SMAD3* KO BT67 cells relative to the AAVS1 control cells. Significance was determined using unpaired two-tailed t-test; *p<0.05; data are represented as fold enrichment mean ± SEM; n=3. Fold enrichment of HDAC2 within the heterochromatin region QML5/6 was used as negative control for ChIP-qPCR. - represents molecular weight markers (50 and 37). Source data are provided in the source data file.

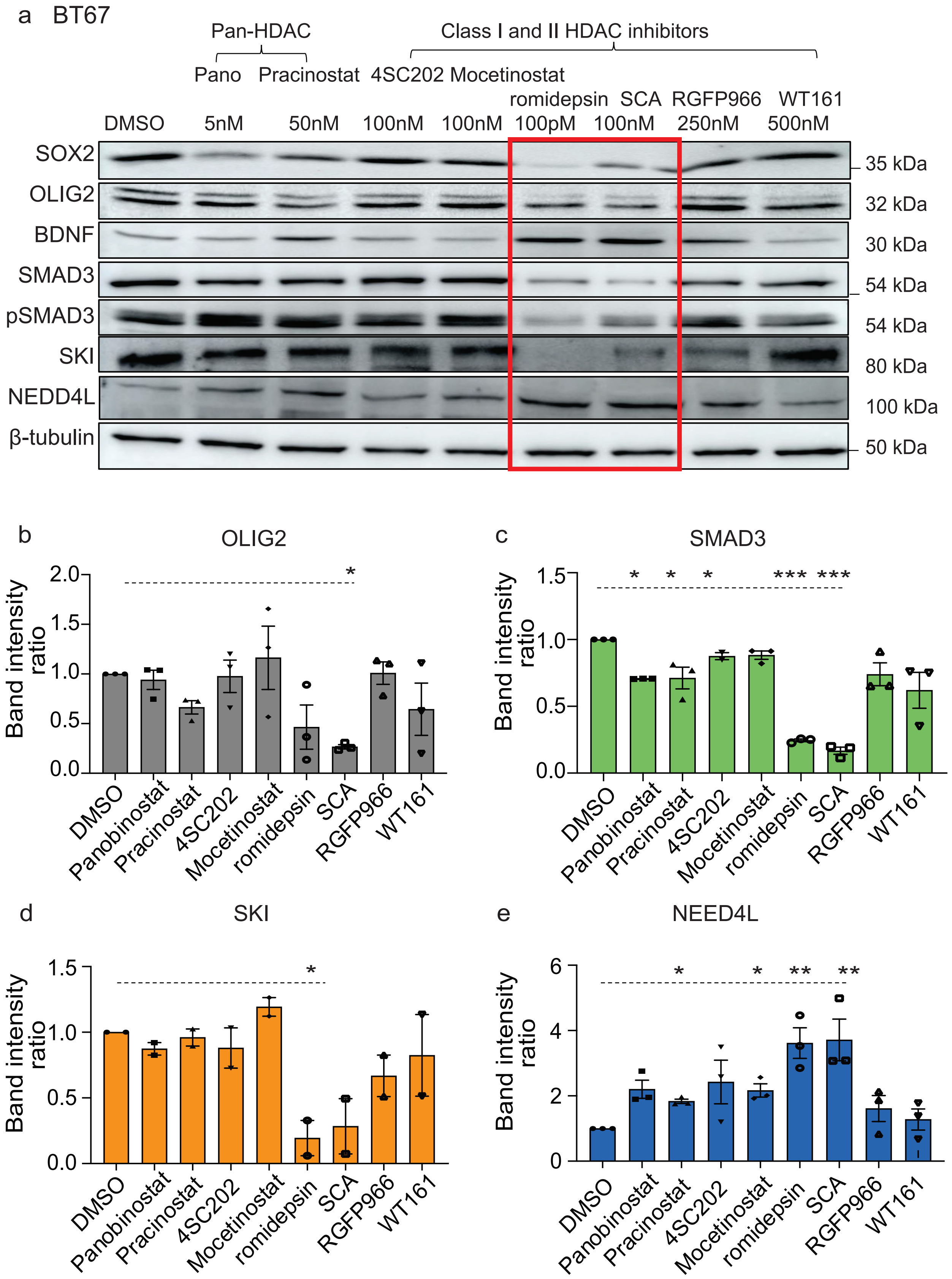

a BT67
Pan-HDAC
Class I and II HDAC inhibitors
Pano Pracinostat 4SC202 Mocetinostat romidepsin SCA RGFP966 WT161
DMSO 5nM 50nM 100nM 100nM 100pM 100nM 250nM 500nM
SOX2
OLIG2
BDNF
SMAD3
pSMAD3
SKI
NEDD4L
β-tubulin
35 kDa
32 kDa
30 kDa
54 kDa
54 kDa
80 kDa
100 kDa
50 kDa
b OLIG2
c SMAD3
d SKI
e NEED4L
Band intensity ratio
DMSO
Panobinostat
Pracinostat
4SC202
Mocetinostat
romidepsin
SCA
RGFP966
WT161

Supplementary Figure 20: a Evaluation of changes in protein levels of stem cell related markers, SOX2 and OLIG2; neuronal cell-fate specific marker BDNF; and TGF-β pathway related proteins, total and phospho-SMAD3, SKI and NEDD4L following treatment with different pan- and class- specific HDAC inhibitors including HDAC2 specific inhibitor Santacruzamate A (SCA) in BT67 cells relative to the vehicle control. n=3. **b-e** Quantifications of band intensity ratios for immunoblots presented in Supp Fig 20a. Significance was determined using ANOVA (Dunnett's test), *$p<0.05$, **$p<0.01$, ***$p<0.001$; data are represented as mean ± SEM, n=3. - represents molecular weight markers (50 and 37). Source data are provided in the source data file.

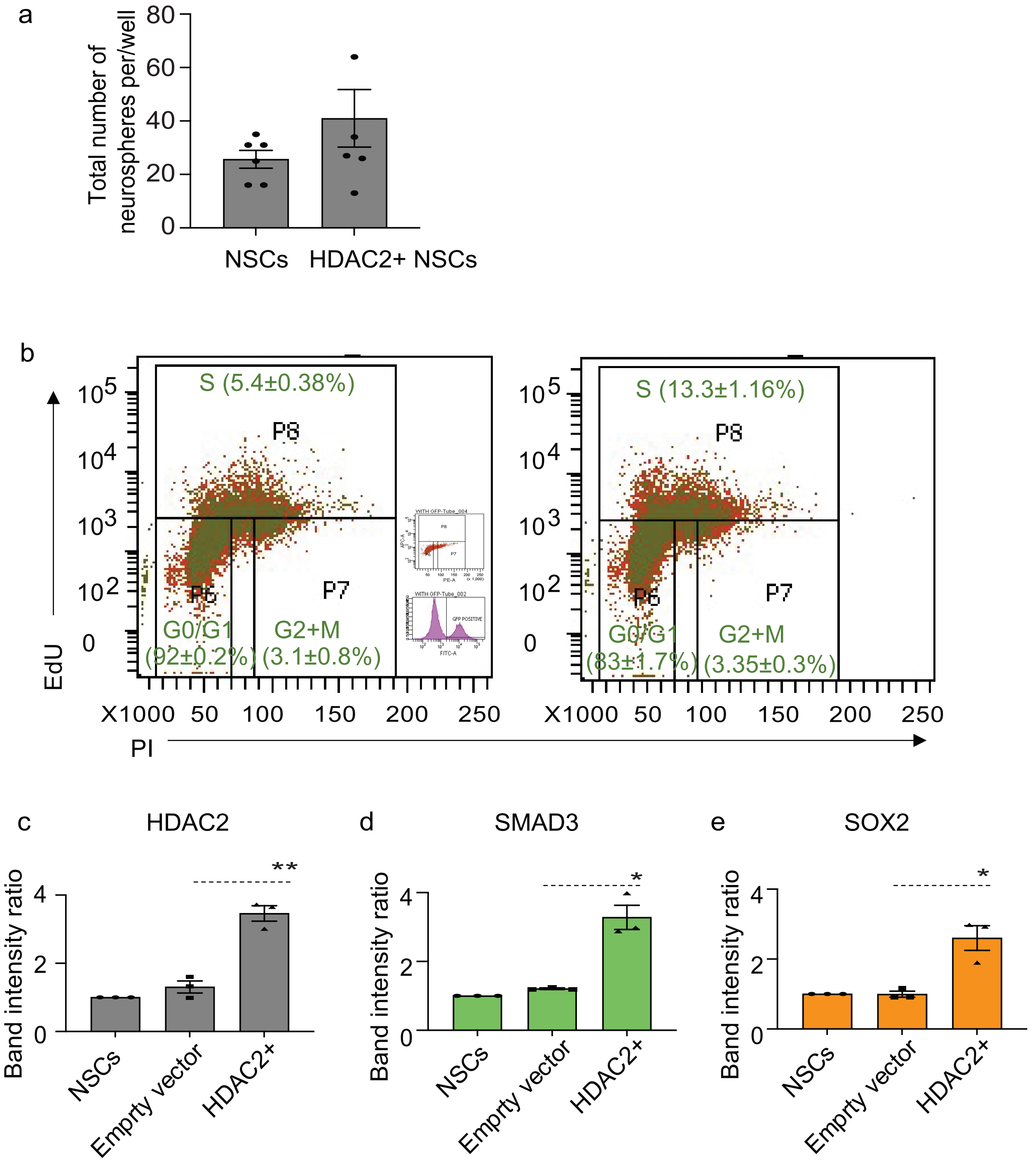
a
Total number of neurospheres per/well
80
60
40
20
0
NSCs
HDAC2+ NSCs
b
S (5.4±0.38%)
P8
P6
P7
G0/G1 (92±0.2%)
G2+M (3.1±0.8%)
S (13.3±1.16%)
G0/G1 (83±1.7%)
G2+M (3.35±0.3%)
EdU
PI
X1000
50
100
150
200
250
c
HDAC2
d
SMAD3
e
SOX2
Band intensity ratio
**
*
NSCs
Emprty vector
HDAC2+

Supplementary Figure 21: a Neurosphere assays determining self-renewal ability of HDAC2+ NSCs and NSCs (empty vector). Significance was determined using unpaired two-tailed t-test, data are represented as mean ± SEM; n=5. **b** Representative flow plots of EdU assays showing the changes in cell cycle progression in $HDAC2^{+}$ NSCs compared to empty vector control cells. Inset shows PI control only and GFP positive cells only. **c-e** Quantification of band intensity ratios for immunoblots presented in Fig 8b. Significance was determined using unpaired two-tailed t-test, *p<0.011, **p<0.0017; data are represented as mean ± SEM, n=3. Gating strategies are provided in Supp. Fig 22a. Source data are provided in the source data file.

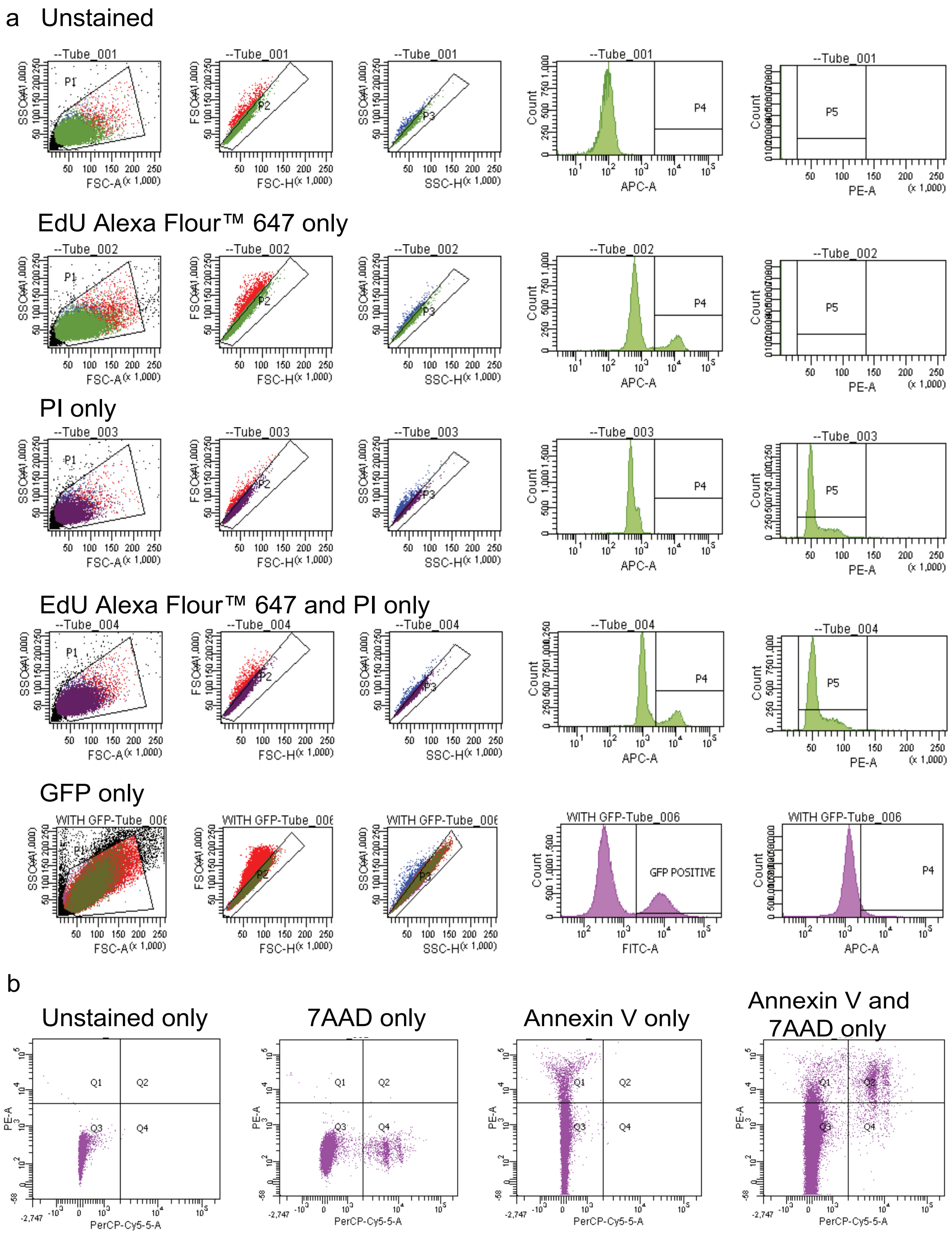
a
Unstained
EdU Alexa Flour™ 647 only
PI only
EdU Alexa Flour™ 647 and PI only
GFP only
--Tube_001
--Tube_002
--Tube_003
--Tube_004
WITH GFP-Tube_006
P1
P2
P3
P4
P5
GFP POSITIVE
FSC-A
FSC-H
SSC-H
APC-A
PE-A
FITC-A
Count
b
Unstained only
7AAD only
Annexin V only
Annexin V and 7AAD only
Q1
Q2
Q3
Q4
PE-A
PerCP-Cy5-5-A

Supplementary Figure 22: Gating strategy used for flow cytometric analysis **a.** EdU cell cycle assays **b.** Annexin V cell survival assays.

Supplementary Tables

Supplementary Table 1: gRNA used in this study.

**gRNA**	**Sequence**
HDAC1-1	GAGCAAGATGGCGCAGACGC
HDAC1-2	CCCTCACAAAGCCAATGCTG
HDAC2-1	ATGGCGTACAGTCAAGGAGG
HDAC2-2	AAACCGACAACAGACTGATA
SMAD3-1	GAAGGGCGAGCAGAACGGGC
SMAD3-2	GAATGGCTGTAGTCGTCCAG
AAVS1	ATCCTGTCCCTAGTGGCCC

Supplementary Table 2: qPCR primers used in this study.

**Gene**	**Forward primer (5' to 3')**	**Reverse primer (5' to 3')**
BDNF	CATAAGGACGCGGACTTGTACA	AGACATGTTTGCGGCATCCA
SOX2	GCTACAGCATGATGCAGGACCA	TCTGCGAGCTGGTCATGGAGTT
SMAD3	GGAAGGGCGTTCTAGGTAGG	CACTGGAAGAGGCACTGTCA
OLIG2	GCTCCTCAAATCGCATCCA	AAAGGTCATCGGGCTCTG
GAPDH	GTGGTCTCCTCTGACTTCAAC	CCTGTTGCTGTAGCCAAATTC

Supplementary Table 3: ChIP-qPCR primers used in this study.

**Gene**	**Forward**	**Reverse**
BDNF set1	GCGGTGGGTGTCTCATTAAA	GAAGCTCAACCGAAGAGCTAAA
BDNF set2	TTCCACGCAGTTCCACATAG	GTTGCTTAACAACGGGCATATC
BDNF PIV	CTGGTAATTCGTGCACTAGAG	CACGAGAGGGCTCCACGGT
SOX2	GTGCTGTTTACCCACTTCCT	GCTCAAACTTCTCTCCCTTTCT
SMAD3	ACGTCAACACCAAGTGCATC	TCCCTCTCTCTCCCTCTTCC
SKI	ACCCCGAAAGACGTCTCAG	GGTGGCTTATCAGGACTTCG
NCAM1	AGGGAGACTGCGTGTGAAAG	GCCGAACATCAAGGAGGTAA
NEDD4L	GGCGCTATGTTGGTTTTGTT	GACGAGCAGGACTTGGCTAC
CDKN1A	CTGGCCTGCTGGAAC TC	CGCTCTCTCACCTCCTCT

Supplementary Table 4: List of primary and secondary antibodies used in this study.

Antibodies	Source	Catalog number	Dilution
Rabbit polyclonal to HDAC1	Abcam	Cat# ab7028	1/2000
Mouse monoclonal to HDAC2	Abcam	Cat# ab12169	1/5000
Rabbit monoclonal to HDAC3	Abcam	Cat# ab7030	1/1000
Rabbit monoclonal to HDAC6	Abcam	Cat# ab82557	1/1000
Rabbit monoclonal to HDAC9 (EPR5223)	Abcam	Cat# ab109446	1/1000
Rabbit monoclonal to SMAD3 (EP568Y)	Abcam	Cat# ab40854	1/2000
Mouse monoclonal to SMAD3	MyBioSource	Cat# MBS5307346	1/1000
Rabbit monoclonal to SMAD3 (phospho S423+S425)	Abcam	Cat# ab52903	1/2000
Rabbit monoclonal to SMAD2 (EP784Y)	Abcam	Cat# ab40855	1/2000
Rabbit polyclonal to MADH7/SMAD7	Abcam	Cat# ab216428	1/1000
Rabbit polyclonal to NEDD4L	Abcam	Cat# ab46521	1/1000
Rabbit polyclonal to SMAD6	Abcam	Cat# ab80049	1/1000
Rabbit polyclonal to SKI	Abcam	Cat# ab19864	1/1000
Rabbit polyclonal to SNON	Abcam	Cat# ab189653	1/2000
Rabbit polyclonal to SIN3A	Abcam	Cat# ab3479	1/1000
Rabbit polyclonal to SOX2	Abcam	Cat# ab97959	1/3000
Rabbit polyclonal to GFAP	Abcam	Cat# ab7260	1/1000
Rabbit monoclonal to STX3 (EPR8543)	Abcam	Cat# ab133750	1/1000
Rabbit monoclonal to BDNF (EPR1292)	Abcam	Cat# ab108319	1/1000
Rabbit monoclonal to p21	Cell signaling	Cat# 2947	1/3000
Rabbit polyclonal to p38	Cell signaling	Cat# 9212	1/3000

Rabbit polyclonal to Beta tubulin	Cell signaling	Cat# 2146	1/3000
Normal rabbit IgG	Millipore Sigma	Cat# 12-370	As per IP antibody
Normal mouse IgG	Millipore Sigma	Cat# 12-371	As per IP antibody
Mouse monoclonal anti-rabbit IgG light chain (HRP)	Abcam	Cat# ab99697	1/2000-1/5000
Rat monoclonal anti-mouse kappa light chain (HRP)	Abcam	Cat# ab99632	1/2000-1/5000
Rabbit monoclonal to H3K27ac (EP16602)	Abcam	Cat# ab177178	1/5000
Rabbit monoclonal to H3K18ac (EP959Y)	Abcam	Cat# ab40888	1/5000
Rabbit polyclonal to H3K56ac	Active motif	Cat# 39082	1/5000
Rabbit monoclonal to H3K9ac	Abcam	Cat# ab 4441	1/5000
Rabbit monoclonal to H3K18Cr (EPR18773)	Abcam	Cat# ab195475	1/5000
Rabbit polyclonal to H3 nuclear marker	Abcam	Cat# ab1791	1/10000
Rabbit monoclonal to H4K5ac (EP1000Y)	Abcam	Cat# ab51997	1/5000
Rabbit monoclonal to H4K16ac (EPR1004)	Abcam	Cat# ab109463	1/5000
Rabbit monoclonal to H4K20ac (EPR16998(2))	Abcam	Cat# ab177191	1/5000
Rabbit polyclonal to H4K12ac	Abcam	Cat# ab46983	1/5000
Rabbit monoclonal to H4 (EPR16599)	Abcam	Cat# 177840	1/5000
Rabbit monoclonal to HDAC2	Selleckchem	Cat# A5000	1/5000
Mouse monoclonal anti-flag M2	Sigma	Cat# F1804	1/1000
