# Supplementary material for: Epigenetic and molecular coordination between HDAC2 and SMAD3-SKI regulates essential brain tumour stem cell characteristics
Source: Nat Commun. 2023 Aug 19;14:5051. doi: 10.1038/s41467-023-40776-y (PMC10439933; doi:10.1038/s41467-023-40776-y)
Supplement: Supplementary file 3 — Description of Additional Supplementary Files [file 41467_2023_40776_MOESM3_ESM.pdf]

Description of additional supplementary files:

File name: Supplementary data 1

Description: romidepsin_treatment_wald_lt_0.05_annotated

File name: Supplementary data 2

Description: QC_sequencing_metrics_2023.xlsx

File name: Supplementary data 3

Description: H4K4ac_HDAC2KO_AAVS1_DEG_results_annotated

File name: Supplementary data 4

Description: HDAC1_HDAC2_SMAD3_peaks_common_anno_counts (1).xlsx

File name: Supplementary data 5

Description: 4-1_MOTIF_enrichment_HDAC2_SMAD3_peaks_common.txt

File name: Supplementary data 6

Description: SMAD3_HDAC2_ Mutant clone sequences: The highlighted regions were used for mutant clone designing.doc
