# Supplementary material for: Epigenetic and molecular coordination between HDAC2 and SMAD3-SKI regulates essential brain tumour stem cell characteristics
Source: Nat Commun. 2023 Aug 19;14:5051. doi: 10.1038/s41467-023-40776-y (PMC10439933; doi:10.1038/s41467-023-40776-y)
Supplement: Supplementary file 10 — Reporting Summary [file 41467_2023_40776_MOESM10_ESM.pdf]

Corresponding author(s): Samuel Weiss / Hema Artee Luchman

Last updated by author(s): Jul 26, 2023

Reporting Summary

Nature Portfolio wishes to improve the reproducibility of the work that we publish. This form provides structure for consistency and transparency in reporting. For further information on Nature Portfolio policies, see our Editorial Policies and the Editorial Policy Checklist.

Statistics

For all statistical analyses, confirm that the following items are present in the figure legend, table legend, main text, or Methods section.

n/a	Confirmed	
☐	☒	The exact sample size (*n*) for each experimental group/condition, given as a discrete number and unit of measurement
☐	☒	A statement on whether measurements were taken from distinct samples or whether the same sample was measured repeatedly
☐	☒	The statistical test(s) used AND whether they are one- or two-sided<br>*Only common tests should be described solely by name; describe more complex techniques in the Methods section.*
☒	☐	A description of all covariates tested
☒	☐	A description of any assumptions or corrections, such as tests of normality and adjustment for multiple comparisons
☐	☒	A full description of the statistical parameters including central tendency (e.g. means) or other basic estimates (e.g. regression coefficient) AND variation (e.g. standard deviation) or associated estimates of uncertainty (e.g. confidence intervals)
☐	☒	For null hypothesis testing, the test statistic (e.g. *F*, *t*, *r*) with confidence intervals, effect sizes, degrees of freedom and *P* value noted<br>*Give P values as exact values whenever suitable.*
☒	☐	For Bayesian analysis, information on the choice of priors and Markov chain Monte Carlo settings
☒	☐	For hierarchical and complex designs, identification of the appropriate level for tests and full reporting of outcomes
☐	☒	Estimates of effect sizes (e.g. Cohen's *d*, Pearson's *r*), indicating how they were calculated

Our web collection on statistics for biologists contains articles on many of the points above.

Software and code

Policy information about availability of computer code

Data collection	IncuCyte ZOOM controller software (Essen BioScience, ver. 2016B), IncuCyteDRC package
Data analysis	Extreme Limiting Dilution Analysis Webtool. GraphPad Prism 8, FACSDiva software version 6.1.3, Qiagen Ingenuity Pathway Analysis (IPA), SuperSignal and Amersham Imager 600 (General Electric), LightCycler 96 software (Roche, ver. 1.1.10.1320), Fiji Image analysis tool ((ImageJ2 v2.14.0), Trimmomatic, MACS2 software suite, HOMER software suite, GESA 4.3.2, Cytoscape version 3, IGV version 2.16.0, findmotifGenome command.

For manuscripts utilizing custom algorithms or software that are central to the research but not yet described in published literature, software must be made available to editors and reviewers. We strongly encourage code deposition in a community repository (e.g. GitHub). See the Nature Portfolio guidelines for submitting code & software for further information.

Data

Policy information about availability of data

All manuscripts must include a data availability statement. This statement should provide the following information, where applicable:

- Accession codes, unique identifiers, or web links for publicly available datasets
- A description of any restrictions on data availability
- For clinical datasets or third party data, please ensure that the statement adheres to our policy

RTNA-seq raw and processed data files for romidepsin treated vs DMSO treated BTSC line BT67 have been deposited at NCBI with accession number: GSE214721
ChIP-seq raw and processed data files for BT67 HDAC1/2 and SMAD3 ChIP-seq and H3K27ac ChIP-seq for romidepsin treated vs DMSO treated BT67 BTSC line and

H4K5ac ChIP-seq for HDAC2KO and AAVS1 control BT67 cells have been deposited at NCBI with accession number: GSE214926. Source data are provided with this manuscript. The remaining data are available within the Article, Supplementary Information or Source Data file provided with this manuscript.

Human research participants

Policy information about studies involving human research participants and Sex and Gender in Research.

Reporting on sex and gender	The characteristics of Glioblastoma (GBM) brain tumour stem cell (BTSC) lines are listed in Table 1.
Population characteristics	GBM BTSC lines were obtained from surgical specimens from patient recruited from the University of Calgary (Calgary, AB, Canada) with written informed consent including sex and age identification.
Recruitment	Patients were recruited following written informed consent.
Ethics oversight	REB HREBA-CC-160762 as approved at the University of Calgary (Calgary, AB, Canada).

Note that full information on the approval of the study protocol must also be provided in the manuscript.

Field-specific reporting

Please select the one below that is the best fit for your research. If you are not sure, read the appropriate sections before making your selection.

☒ Life sciences ☐ Behavioural & social sciences ☐ Ecological, evolutionary & environmental sciences

For a reference copy of the document with all sections, see nature.com/documents/nr-reporting-summary-flat.pdf

Life sciences study design

All studies must disclose on these points even when the disclosure is negative.

Sample size	No sample size calculation were performed for the experiments conducted. The sample size for each experiment is provided in the figure legends and in the main manuscript. Sample sizes were chosen to enable confident and meaningful conclusions from each experimental outcome. Sample sizes of N=3 Biological replicates were used to determine significant differences in the changes observed in analyzed biological assays. For experiments with high level of variability, such as determining the dose responses to drugs, as many as possible BTSC lines were tested with a maximum of 6 technical replicates within each biological replicate. For in vivo experiments, at least 8 -12mice/ group were used to assess changes in animal survival. No formal sample size calculation was performed for in vivo experiments, in order to generate the Kaplan-Meier survival data, 10-12mice/ group were used to account for effect size for mean difference between experimental groups, power of the study at 80% and adjusted sample size to account for attrition from up to 10% cell death from unexpected events.
Data exclusions	For in vivo experiments in Fig 4a, b, Fig 6e, Supp Fig 13e and Supp Fig 19b, the animals who died during the experiment due to unexplained reasons were removed from the study. For quantifications of immunoblots, the replicate with high background signal were impacting the correct assessment of band intensities and were removed from the analyses.
Replication	All in vitro / molecular experiments were performed using n=3 biologicals replicates on two independent BTSC lines. For experiments with high level of variability, such as determining the dose responses to drugs, as many as possible BTSC lines were tested with a maximum of 6 technical replicates within each biological replicate. For CRISPR-Cas9 knockout 2 individual guide RNA sequences along with AAVS1 cut control and for shRNA knockdown 3 independent shRNA sequences along with their respective scrambled control were used for each gene. All findings were reproducible over several experiments. For RNA-seq and ChIP-seq experiments. the n=3 biologically independent samples were collected for sequencing and for qPCR and ChIP-qPCR studies.
Randomization	Randomization was not feasible for the in vitro studies however, for in vivo experiments, mice xenotransplanted with knockout/knockdown vs control BTSC line were randomly houed in cages.
Blinding	All the in vivo experiments were blinded to the individual who performed the surgeries in mice. For LDA experiments, the scoring was performed by 2 independent observers to minimize bias or ensure reproducibility. Remaining studies were not blinded but were replicated using independent methods and using additional cell line.

Reporting for specific materials, systems and methods

We require information from authors about some types of materials, experimental systems and methods used in many studies. Here, indicate whether each material, system or method listed is relevant to your study. If you are not sure if a list item applies to your research, read the appropriate section before selecting a response.

Materials & experimental systems

n/a	Involved in the study
	☒ Antibodies
	☒ Eukaryotic cell lines
☒	Palaeontology and archaeology
	☒ Animals and other organisms
☒	Clinical data
☒	Dual use research of concern

Methods

n/a	Involved in the study
	☒ ChIP-seq
	☒ Flow cytometry
☒	MRI-based neuroimaging

Antibodies

Antibodies used

Antibodies Source Catalog number Dilution
Rabbit polyclonal to HDAC1 Abcam Cat# ab7028 1/2000
Mouse monoclonal to HDAC2 Abcam Cat# ab12169 1/5000
Rabbit monoclonal to HDAC3 Abcam Cat# ab7030 1/1000
Rabbit monoclonal to HDAC6 Abcam Cat# ab82557 1/1000
Rabbit monoclonal to HDAC9 (EPR5223) Abcam Cat# ab109446 1/1000
Rabbit monoclonal to SMAD3 (EP568Y) Abcam Cat# ab40854 1/2000
Mouse monoclonal to SMAD3 MyBioSource Cat# MBS5307346 1/1000
Rabbit monoclonal to SMAD3 (phospho S423+S425) Abcam Cat# ab52903 1/2000
Rabbit monoclonal to SMAD2 (EP784Y) Abcam Cat# ab40855 1/2000
Rabbit polyclonal to MADH7/SMAD7 Abcam Cat# ab216428 1/1000
Rabbit polyclonal to NEDD4L Abcam Cat# ab46521 1/1000
Rabbit polyclonal to SMAD6 Abcam Cat# ab80049 1/1000
Rabbit polyclonal to SKI Abcam Cat# ab19864 1/1000
Rabbit polyclonal to SNON Abcam Cat# ab189653 1/2000
Rabbit polyclonal to SIN3A Abcam Cat# ab3479 1/1000
Rabbit polyclonal to SOX2 Abcam Cat# ab97959 1/3000
Rabbit polyclonal to GFAP Abcam Cat# ab7260 1/1000
Rabbit monoclonal to STX3 (EPR8543) Abcam Cat# ab133750 1/1000
Rabbit monoclonal to BDNF (EPR1292) Abcam Cat# ab108319 1/1000
Rabbit monoclonal to p21 Cell signaling Cat# 2947 1/3000
Rabbit polyclonal to p38 Cell signaling Cat# 9212 1/3000
Rabbit polyclonal to Beta tubulin Cell signaling Cat# 2146 1/3000
Normal rabbit IgG Millipore Sigma Cat# 12-370 As per IP antibody
Normal mouse IgG Millipore Sigma Cat# 12-371 As per IP antibody
Mouse monoclonal anti-rabbit IgG light chain (HRP) Abcam Cat# ab99697 1/2000-1/5000
Rat monoclonal anti-mouse kappa light chain (HRP) Abcam Cat# ab99632 1/2000-1/5000
Rabbit monoclonal to H3K27ac (EP16602) Abcam Cat# ab177178 1/5000
Rabbit monoclonal to H3K18ac (EP959Y) Abcam Cat# ab40888 1/5000
Rabbit polyclonal to H3K56ac Active motif Cat# 39082 1/5000
Rabbit monoclonal to H3K9ac Abcam Cat# ab 4441 1/5000
Rabbit monoclonal to H3K18Cr (EPR18773) Abcam Cat# ab195475 1/5000
Rabbit polyclonal to H3 nuclear marker Abcam Cat# ab1791 1/10000
Rabbit monoclonal to H4K5ac (EP1000Y) Abcam Cat# ab51997 1/5000
Rabbit monoclonal to H4K16ac (EPR1004) Abcam Cat# ab109463 1/5000
Rabbit monoclonal to H4K20ac (EPR16998(2)) Abcam Cat# ab177191 1/5000
Rabbit polyclonal to H4K12ac Abcam Cat# ab46983 1/5000
Rabbit monoclonal to H4 (EPR16599) Abcam Cat# 177840 1/5000
Rabbit monoclonal to HDAC2 Selleckchem Cat# A5000 1/5000
Mouse monoclonal anti-flag M2 Sigma Cat# F1804 1/1000

Validation

All antibodies were used as per Manufacturer's guidelines and are cited in the literature. Western blots using human GBM BTSC lysates from romidepsin treated vs DMSO treated cells and from HDAC1/2 knockout/knockdown vs control BT67 and BT147 cells were used to determine changes in the protein levels of target proteins.

Eukaryotic cell lines

Policy information about cell lines and Sex and Gender in Research

Cell line source(s)

All the used BTSC lines were derived from surgical specimens, following written informed consent for disclosure from patients with GBM and their characteristics are listed in main Table 1. Human induced pluripotent stem cells (hiPSCs) and HEK293T/17 cells were obtained from ATCC and Normal human fetal neural (HF-NSCs) stem cells were established from human fetal tissue.

Authentication

All GBM BTSC lines were authenticated using short tandem repeat (STR) profiling and their profile was compared to the original parental tumors tissue (Calgary Laboratory Services and Department of Pathology and Laboratory Medicine,

University of Calgary). Authentication and testing of all cell lines was performed as per American Association for Cancer Research recommendations.

Mycoplasma contamination

All cell lines used in this study were routinely tested for Mycoplasma using Vendor™ GeM Mycoplasma PCR-based detection kit (Sigma, MP0025) and they were confirmed to be mycoplasma free.

Commonly misidentified lines
(See ICLAC register)

No cell line utilized in our study are listed in the ICLAC register.

Animals and other research organisms

Policy information about studies involving animals; ARRIVE guidelines recommended for reporting animal research, and Sex and Gender in Research

Laboratory animals

6-8 weeks old CB-17 female SCID mice were housed in Biohazard barrier levels 2 facility at a temperature 25+/-2C, 45-55% humidity, and a light cycle of 6am on, 8pm off.

Wild animals

No wild animals were used in the study.

Reporting on sex

All animals used were female, and as such no sex-based analysis was performed. Female mice were used for ease of housing purposes

Field-collected samples

as per our animal animal ethics guidelines as adult male mice show aggressiveness in communal situations and have to housed

Ethics oversight

separately which further creates under stress for mice and requires additional housing space within the facility.

No field collected samples were used in the study.

Note that full information on the approval of the study protocol must also be provided in the manuscript.

All animal procedures were performed according to animal ethics protocol, approved by the Animal Care Committee of the University of Calgary and operating under the Guidelines of the Canadian Council of Animal Care.

ChIP-seq

Data deposition

- [x] Confirm that both raw and final processed data have been deposited in a public database such as GEO.
- [x] Confirm that you have deposited or provided access to graph files (e.g. BED files) for the called peaks.

Data access links
May remain private before publication.

https://www.ncbi.nlm.nih.gov/geo/query/acc.cgi?acc=GSE214721

https://www.ncbi.nlm.nih.gov/geo/query/acc.cgi?acc=GSE214926

Files in database submission

GSM6614911 BT67_DMSO_1
GSM6614912 BT67_romidepsin_1
GSM6614913 BT67_DMSO_2
GSM6614914 BT67_romidepsin_2
GSM6614915 BT67_DMSO_3
GSM6614916 BT67_romidepsin_3

GSM7434700 BT67_HDAC1_1
GSM7434701 BT67_HDAC1_2
GSM7434702 BT67_HDAC1_3
GSM7434703 BT67_HDAC2_1
GSM7434704 BT67_HDAC2_2
GSM7434705 BT67_HDAC2_3
GSM7434706 BT67_SMAD3_1
GSM7434707 BT67_SMAD3_2
GSM7434708 BT67_SMAD3_3
GSM7434709 BT67_Input_1
GSM7434710 BT67_Input_2
GSM7434711 BT67_Input_3
GSM6656031 BT67_H3K27ac_DMSO_1
GSM6656032 BT67_H3K27ac_DMSO_2
GSM6656033 BT67_H3K27ac_DMSO_3
GSM6656034 BT67_H3K27ac_romi_1
GSM6656035 BT67_H3K27ac_romi_2
GSM6656036 BT67_H3K27ac_romi_3
GSM6656037 BT67_H3K27ac_DMSO_Input_1
GSM6656038 BT67_H3K27ac_DMSO_Input_2
GSM6656039 BT67_H3K27ac_DMSO_Input_3
GSM6656040 BT67_H3K27ac_romi_Input_1
GSM6656041 BT67_H3K27ac_romi_Input_2
GSM6656042 BT67_H3K27ac_romi_Input_3
GSM7434712 BT67_HDAC2KO_H4K5ac_1

GSM7434713 BT67_HDAC2KO_H4K5ac_2
GSM7434714 BT67_HDAC2KO_H4K5ac_3
GSM7434715 BT67_AAVS1_H4K5ac_1
GSM7434716 BT67_AAVS1_H4K5ac_2
GSM7434717 BT67_AAVS1_H4K5ac_3
GSM7434718 BT67_Input_1
GSM7434719 BT67_Input_2
GSM7434720 BT67_Input_3

Genome browser session (e.g. UCSC)	http://genome.ucsc.edu/s/apacis/ChIPseq_Ravinder_Bahia

Methodology

Replicates	N=3 biological replicates were used
Sequencing depth	Novaseq SP 200 cycle V1.5, 800M read pairs, 167Gbp for ChIP-seq
Antibodies	Rabbit polyclonal to HDAC1 Abcam Cat# ab7028, Mouse monoclonal to HDAC2 Abcam Cat# ab12169, Rabbit monoclonal to SMAD3 (EP568Y) Abcam Cat# ab40854, Normal rabbit IgG Millipore Sigma Cat# 12-370, Normal mouse IgG Millipore Sigma Cat# 12-371, Rabbit monoclonal to H3K27ac (EP16602) Abcam Cat# ab177178, Rabbit monoclonal to H4K5ac (EP1000Y) Abcam Cat# ab51997
Peak calling parameters	Peaks were called using MACS2 software suite. A "reference peak set" was obtained by merging ChIP-seq peaks from each samples using bed tools merge with parameters: -sorted -d -125 (https://bedtools.readthedocs.io/). Peaks were associated with the nearest TSS of genes (+/- 20kb) using the annotate Peaks command from HOMER software suite. Peak enrichments were calculated as Fragments Per Kilobase of transcript per Million mapped reads (FPKM).
Data quality	ChIP-seq reads were first trimmed for adapter sequences and low quality score bases using Trimmomatic. Only reads that had a unique alignment (mapping quality > 20) were retained and PCR duplicates were marked using Picard tools (https://broadinstitute.github.io/picard/).
Software	Nova 6000, Trimmomatic, MACS2 software suite, HOMER software suite

Flow Cytometry

Plots

Confirm that:

- [x] The axis labels state the marker and fluorochrome used (e.g. CD4-FITC).
- [x] The axis scales are clearly visible. Include numbers along axes only for bottom left plot of group (a 'group' is an analysis of identical markers).
- [x] All plots are contour plots with outliers or pseudocolor plots.
- [x] A numerical value for number of cells or percentage (with statistics) is provided.

Methodology

Sample preparation	Treated vs. untreated BTSC lines
Instrument	CytoFLEX LX (Beckman Coulter)
Software	FACSDiva software version 6.1.3 (BD Biosciences)
Cell population abundance	Annexin V+/7AAD- and Annexin V+/7AAD+ cells were analyzed for Cell survival assays. EdU Alexa Fluor™ 647 and PI stained cells were analyzed for EdU cell cycle analysis.
Gating strategy	Unstained, single stained and double stained cell controls were used to set gates and to eliminate negative cell population for each experiment. A supplementary figure about Gating strategy has been submitted along with summary report.

- [x] Tick this box to confirm that a figure exemplifying the gating strategy is provided in the Supplementary Information.
